# Leveraging carrier mobility enables high-performance Mg_3_(Sb, Bi)_2_ thermoelectrics

**DOI:** 10.1093/nsr/nwaf507

**Published:** 2025-11-17

**Authors:** Longquan Wang, Airan Li, Xinzhi Wu, Jiankang Li, Takao Mori

**Affiliations:** Research Center for Materials Nanoarchitectonics (MANA), National Institute for Materials Science (NIMS), Tsukuba 305-0044, Japan; Research Center for Materials Nanoarchitectonics (MANA), National Institute for Materials Science (NIMS), Tsukuba 305-0044, Japan; Research Center for Materials Nanoarchitectonics (MANA), National Institute for Materials Science (NIMS), Tsukuba 305-0044, Japan; Research Center for Materials Nanoarchitectonics (MANA), National Institute for Materials Science (NIMS), Tsukuba 305-0044, Japan; Graduate School of Pure and Applied Sciences, University of Tsukuba, Tsukuba 305-8671, Japan; Research Center for Materials Nanoarchitectonics (MANA), National Institute for Materials Science (NIMS), Tsukuba 305-0044, Japan; Graduate School of Pure and Applied Sciences, University of Tsukuba, Tsukuba 305-8671, Japan

**Keywords:** thermoelectric, carrier scattering, carrier mobility, Mg_3_(Sb, Bi)_2_, thermoelectric device

## Abstract

Carrier transport critically governs the thermoelectric performance of semiconductors, but its optimization remains challenging due to the coexistence of multiple scattering mechanisms. Herein, we construct a mobility diagram for Mg_3_(Sb, Bi)_2_ by capturing the effects of acoustic-phonon, grain-boundary and polar-optical-phonon scattering to guide targeted optimization. This approach enables a top-tier carrier mobility of 179 cm^2^ V^−1^ s^−1^ in this material system. The exceptional transport properties yield a peak figure of merit (*zT*) of ∼2.0 at 723 K and an average *zT* of 1.4 over the range of 300–723 K. These material-level improvements translate into outstanding device performance: a single-leg module reaches ∼13% conversion efficiency and a fully Mg-based two-pair module achieves ∼8% under a temperature difference of 297 K. These findings highlight not only the high potential of Mg_3_(Sb, Bi)_2_ for efficient power generation, but also the pivotal role of carrier transport as a design metric in thermoelectric materials.

## Teaser

Constructing a carrier-mobility diagram clarifies scattering mechanisms and guides the realization of high-performance Mg₃(Sb,Bi)₂ thermoelectrics.

## INTRODUCTION

Accelerating the transition toward carbon neutrality demands technologies capable of converting ubiquitous waste heat into usable energy [1]. Yet, according to the second law of thermodynamics, thermal losses are inevitable in virtually all energy systems, underscoring the critical need for efficient waste-heat recovery. Thermoelectric (TE) technology offers a solid-state solution by directly converting thermal gradients into electricity [2], aligning with global climate goals such as the United Nations Sustainable Development Goals. The energy-conversion efficiency of TE technology hinges on the material’s dimensionless figure of merit, *zT* = *S*^2^*σT*/(*κ*_lat_  *+ κ*_ele_), where *S, σ, T, κ*_lat_ and *κ*_ele_ are the Seebeck coefficient, electrical conductivity, absolute temperature, lattice thermal conductivity and electronic thermal conductivity, respectively [3, 4]. These interdependent parameters reflect the intricate balance between charge and heat transport. To decouple this trade-off, the TE quality factor *B* is often used as a predictor of achievable *zT* [5] and is proportional to *μ*(m*)^3/2^/*κ*_lat_, where *μ* is the carrier mobility and *m*^⁎^ is the effective mass. While extensive efforts—such as defect engineering and nanostructuring [6–9]—have been devoted to suppressing *κ*_lat_, they often inadvertently impair *μ* by introducing additional scattering centers. Thus, a fundamental understanding and rational modulation of *μ* represent a compelling strategy in the development of high-efficiency TE materials.

**Figure 1.**
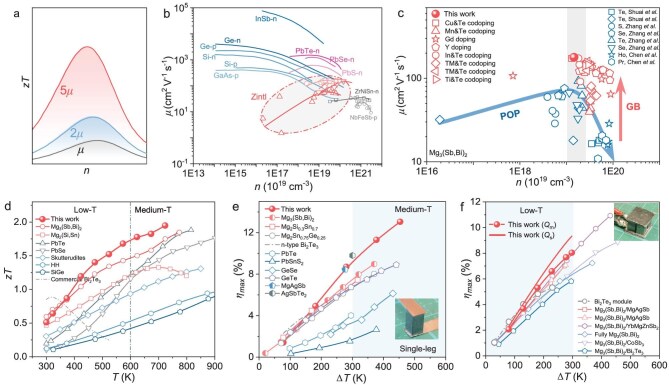
High TE performance in Mg_3_(Sb, Bi)_2_ via *μ* optimization. (a) Schematic illustration of *zT* variation as a function of *n* and *μ* in TE materials, highlighting the role of *μ* in enhancing *zT*. (b) *μ* versus *n* at room temperature in typical semiconductors, with data summarized from the literature [18–22]. (c) *n*-dependent *μ* in Mg_3_(Sb, Bi)_2_ based on data from this work and literature, with the arrows serving as a visual reference. (d) Temperature-dependent *zT* values of the optimized Mg_3_(Sb, Bi)_2_ compared with those representative TE materials (Table S2). Conversion efficiency of the (e) single-leg and (f) two-pair Mg-based modules compared with the literature [31–37].

In TE materials, *μ* determines how efficiently charge carriers contribute to electrical conduction and hence plays a crucial role in optimizing both the power factor (*PF* = *S*^2^*σ*) and the overall *zT* (Fig. 1a) [3, 4]. Conventional *PF* optimization involves a trade-off between *S* and *σ* through carrier concentration (*n*) tuning [3]. However, as *μ* directly contributes to *σ* (*σ* = *n*e*μ*), its enhancement can improve electrical transport efficiency, offering an avenue to boost *PF* within the same doping regime. Various recent approaches—including atomic ordering, structural modulation and deformation potential engineering [10–14]—have demonstrated the potential to enhance *μ* and improve TE performance. These successful strategies underscore the importance of understanding the fundamental factors that govern carrier transport. Under the assumption of elastic scattering and a simple parabolic band, *μ* is expressed as *μ* = *eτ*/m* [15], where *τ* is the relaxation time. Accordingly, reducing m* or tailoring *τ* through scattering mechanisms offer promising pathways for *μ* enhancement. However, a reduced m* often leads to a lower *S*, which limits *PF* gains. Therefore, manipulating *τ* via selective scattering processes emerges as a more effective strategy for boosting *μ*—and thereby improving TE performance.

Among the various scattering mechanisms, acoustic-phonon scattering, originating from thermal vibrations of the lattice [4], is typically dominant at intermediate temperatures. However, additional scattering sources—including the grain boundary, defects and polar optical phonons—can significantly influence carrier transport, particularly in structurally complex compounds. Each of these processes exhibits distinct dependencies on parameters such as temperature, effective mass and carrier energy [15], offering tunable pathways to enhance *μ*. For instance, the suppression of grain-boundary scattering has led to improved low-temperature *μ* in PbTe [16], InTe [12] and Mg_3_(Sb, Bi)_2_ [17]. Likewise, polar-optical-phonon scattering, which severely limits *μ* in Zintl and half-Heusler compounds [18, 19], can be alleviated via enhanced dielectric screening through targeted doping. Thus, elucidating the relative contributions of these coexisting scattering mechanisms is crucial for constructing a comprehensive mobility diagram that can systematically guide *μ*-enhancement strategies across the entire doping regime.

Although temperature-dependent *μ* behavior is a common diagnostic for scattering mechanisms, it often reflects a convoluted effect of multiple interactions. In contrast, analysing the *n*–*μ* relationship at constant temperature serves as a more resolvable indicator for identifying transitions in scattering regimes. This is because tuning *n* effectively adjusts the Fermi level and hence the carrier energy, which strongly influences the scattering probabilities. Thus, constructing a *n–μ* diagram is particularly powerful, not only for unraveling the scattering mechanisms, but also for guiding doping strategies to optimize carrier transport and TE performance. In typical semiconductors, such as Si, Ge and InSb, where acoustic-phonon scattering dominates, *μ* decreases monotonically with increasing *n* (Fig. 1b) [18]. In contrast, a non-monotonic trend in *μ* has been observed in materials such as PbTe/Se/S and half-Heusler compounds [18, 20–22], indicating transitions in scattering mechanisms. In Zintl compounds, anomalously low *μ* at low *n* has recently been attributed to strong polar-optical-phonon scattering [19], further illustrating the diagnostic power of the *n–μ* framework.

As a state-of-the-art TE material, Mg_3_(Sb, Bi)_2_ combines a favorable electronic structure, mechanical robustness and earth-abundant constituents [23, 24]. However, despite these intrinsic advantages, its *μ*—and thus *zT*—is often compromised by complex scattering environments. Grain-boundary scattering has been widely recognized as a key bottleneck [25], mitigated through microstructural engineering such as grain coarsening and interface manipulating [26–28]. Yet, defect-induced scattering remains under debate [29] and, more critically, strong polar-optical-phonon scattering arising from the polar-covalent Mg–Sb bonds poses an intrinsic limitation [30]. In this context, constructing a systematic *n*–*μ* diagram provides a powerful framework for understanding these intertwined scattering contributions and to guide rational strategies in doping, alloying and microstructural tuning. Such an approach is critical to unlocking the full performance potential of Mg_3_(Sb, Bi)_2_.

In this work, we construct an *n*–*μ* diagram to elucidate the complex carrier-scattering processes in Mg_3_(Sb, Bi)_2_, encompassing acoustic-phonon, defect, grain-boundary and polar-optical-phonon scattering. Guided by this framework, we rationally design the carrier-transport behavior, achieving a high *μ* of 179 cm^2^ V^−1^ s^−1^, representing one of the most favorable values in this system. Concurrently, the *κ*_lat_ is effectively suppressed, resulting in an enhanced quality factor *B* and a peak *zT* of ∼2.0 at 723 K. The optimized composition further enables outstanding module-level performance, with a conversion efficiency of ∼13% for a single-leg and ∼8% for a two-pair Mg-based module under a moderate temperature gradient (453/297 K). These results not only highlight the practical potential of Mg_3_(Sb, Bi)_2_-based thermoelectrics, but also offer an effective strategy for tailoring carrier scattering in TE materials.

## RESULTS AND DISCUSSION

### Achievement of high performance in Mg_3_(Sb, Bi)_2_ via maximizing ***μ***

As shown in Fig. 1c, the compiled *μ* as a function of *n* reveals a distinct non-monotonic trend in Mg_3_(Sb, Bi)_2_, based on both literature data and our own results (Table S1). The suppressed *μ* in the low-*n* region is mainly attributed to polar-optical-phonon scattering, which will be further discussed later. The decrease in *μ* at high *n* can be understood by acoustic-phonon scattering, in which changed carrier energy or changes in the density of states near the Fermi level play significant roles. Possible defect-related scattering may also contribute to this trend [29]. Notably, the data can be categorized into two groups (blue and red points), with the transition associated with modified grain-boundary characteristics via doping or alloying. This enhancement is attributed to the weakened grain-boundary scattering—a phenomenon well established in Mg_3_(Sb, Bi)_2_ [25]. Based on this summarized *n*–*μ* diagram, we engineered samples with optimized *n* and enlarged grain size via tailored doping and synthesis strategies, ultimately achieving an ultra-high *μ* of 179 cm^2^ V^−1^ s^−1^.

Benefitting from the significantly enhanced *μ*, the optimized Mg_3_(Sb, Bi)_2_ samples exhibit outstanding TE performance, with a *zT* of ∼0.5 at room temperature and ∼2.0 at 723 K—surpassing most conventional TE materials (Fig. 1d). These results underscore the excellent potential of Mg_3_(Sb, Bi)_2_ for thermal energy harvesting over a broad temperature range. To verify the practical applicability, a single-leg TE module was fabricated by using the optimized material, achieving a remarkable conversion efficiency of ∼13% under a temperature difference of 453 K (Fig. 1e). Compared with other single-leg modules (Table S2), it shows clear advantages in mid-temperature power generation. Furthermore, a two-pair Mg-based module composed of n-type Mg_3_(Sb, Bi)_2_ and p-type MgAgSb exhibited excellent performance for low-grade heat recovery, delivering a measured efficiency of ∼8% under a temperature gradient of 297 K (Fig. 1f). Simulated heat-flow calculations further suggest a corrected efficiency of 9.3%. Notably, both the measured (∼8%) and corrected (9.3%) efficiencies exceed those of most previously reported two-pair modules targeting low-grade heat recovery (Δ*T* < 300 K) [31–37]. These results demonstrate the great promise of Mg_3_(Sb, Bi)_2_-based modules for efficient power generation from waste heat.

### Manipulating carrier transport via scattering mechanisms

To systematically investigate the carrier-transport behavior in Mg_3_(Sb, Bi)_2_, we employed a targeted doping strategy and microstructure optimization guided by the *n*–*μ* map. Sn was intentionally introduced at the Sb site—not only to modify the *n* and mitigate acoustic-phonon scattering, but also to potentially weaken polar-optical-phonon scattering. The latter effect is hypothesized based on the reduced electronegativity difference between Sn and Sb, which may weaken the bond polarity and hence diminish long-range polar interactions. In addition, Sn doping may promote partial liquid-phase sintering due to its relatively low melting point, thereby facilitating grain growth and reducing grain-boundary scattering. To validate these hypotheses, a series of Sn-doped Mg_3_(Sb, Bi)_2_ samples were synthesized with varying doping levels and selected compositions were subjected to prolonged sintering to further promote grain growth. X-ray diffraction patterns confirm phase purity with no secondary phase detected and the refined lattice parameters remain nearly constant (Fig. S1). Scanning electron microscopy (SEM) and energy-dispersive X-ray spectroscopy (EDS) mapping on polished surfaces reveal uniform elemental distribution and no Sn-rich secondary phases (*x* = 0.02-T20), indicating that the Sn solubility limit is at least *x* = 0.02 (Fig. S2). The nearly unchanged lattice parameters and uniform elemental distribution indicate that Sn preferentially occupies the Sb sites, consistently with the similar atomic radius and chemical properties of Sn and Sb.

**Figure 2.**
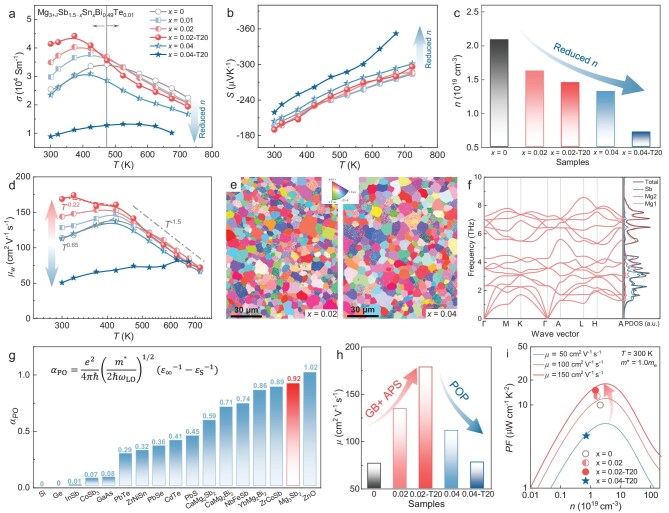
Enhanced *μ* through manipulation of carrier-scattering mechanisms. Temperature dependence of (a) *σ* and (b) *S*. (c) Room-temperature Hall *n*. (d) Temperature-dependent *μ*_w_. (e) Electron backscatter diffraction crystal-orientation maps of *x* = 0.02 and *x* = 0.04 samples. (f) Phonon dispersion and phonon density-of-state (PDOS) of Mg_3_Sb_2_. (g) Calculated *α*_PO_ for typical semiconductors and TE materials [18, 19, 43]; parameters for Mg_3_Sb_2_ are listed in Table S3. (h) Room-temperature *μ* of the samples. (i) Experimental *PF* and single-parabolic-band-model-simulated *PF* (lines) as a function of *n*.

Guided by the insights from the *n*–*μ* map, we next characterized the temperature-dependent transport behaviors of Sn-doped samples. The temperature-dependent *σ* of the samples can be divided into two regimes (Fig. 2a). At low temperatures (*T* < 473 K), *σ* first increases and then decreases with doping, suggesting a change in the dominant carrier-scattering mechanism. In contrast, at higher temperatures (*T* > 473 K), *σ* decreases monotonically while *S* increases correspondingly (Fig. 2b), mainly due to a reduction in *n* with doping. Hall measurements further confirm this variation and a continually decreased *n* has been detected in the samples (Fig. 2c). The monotonic decrease in *n* with increasing Sn content arises from the acceptor behavior of Sn substituting Sb sites and the extended sintering further reduces *n* due to Mg evaporation. The unusual behavior of *σ* at low temperatures suggests a shift in carrier-scattering mechanisms, which will be explored in the following sections.

Carrier-transport behavior was quantitatively assessed through the weighted mobility *μ*_w_ [5], derived from measured *σ* and *S*, as shown in Fig. 2d. At high temperatures, *μ*_w_ follows a *T*^−1.5^ dependence, consistently with dominant acoustic-phonon scattering. In contrast, thermally activated behavior is observed at low temperatures, typically associated with grain-boundary scattering in polycrystalline Mg_3_(Sb, Bi)_2_ [25]. Upon targeted Sn doping and extended sintering (*x* = 0.02-T20), *μ*_w_ markedly increases, reaching 169 cm^2^ V^−1^ s^−1^ at 300 K—representing a 48% enhancement over the undoped sample. The increase in *μ*_w_ and the stronger temperature dependence at low temperatures are consistent with suppressed grain-boundary scattering, enabled by microstructural evolution. Sn doping leads to slight grain coarsening (from 4.4 to 5.25 μm, Fig. S3), likely due to local melting-point depression [38], and prolonged sintering further promotes thermally driven grain growth, as supported by electron backscatter diffraction and fracture morphology analyses (Fig. 2e and Fig. S4). Grain-boundary features, in addition to grain size, can also influence carrier scattering. However, given the low melting point of Sn and its preferential substitution at Sb sites, Sn doping is unlikely to significantly alter grain-boundary chemistry or form metallic boundary phases. Besides, defect-related scattering may affect carrier transport, but its impact appears to be limited in the present samples.

Beyond grain-boundary effects, acoustic-phonon scattering is also mitigated. As *μ*_w_ ≈ *μ*(m*/*m*_e_)^3/2^, a decrease in m* makes *μ*_w_ grow more slowly than *μ*; hence, improvements in carrier transport may not be fully captured by *μ*_w_ relative to *μ*. Single-parabolic-band (SPB) fitting indicates a reduction in the m* in our samples (Fig. S5) and first-principles calculations confirm that Sn substitution at the Sb site slightly shifts the Fermi level and reduces the density-of-state (DOS) m*, consistently with the experimental results (Fig. S6). In this fitting, the scattering factor was assumed to follow acoustic-phonon-dominated behavior and the observed changes in the *S* mainly reflect the decrease in m*. In our optimized samples, the reduction in *n* and m* weakens electron–phonon coupling and suppresses acoustic-phonon scattering by lowering the density of thermally active carriers and reducing the scattering rates. The concurrent reduction in both the intrinsic (acoustic-phonon) and extrinsic (grain-boundary) scattering mechanisms thus underpins the enhanced carrier transport in the optimized samples.

In contrast, *μ*_w_ is markedly reduced in the *x* = 0.04 and *x* = 0.04-T20 samples, despite further reductions in *n* and an enlarged grain size. This behavior cannot be explained solely by conventional scattering mechanisms and suggests a transition to a new dominant scattering source. Given the reduced *n* and the polar nature of Mg_3_(Sb, Bi)_2_, polar-optical-phonon scattering is expected to become significant. The origin of polar-optical-phonon scattering lies in the dipole moments generated by oppositely charged atoms in polar materials [39]. These dipoles oscillate under longitudinal optical (LO) phonon vibrations, creating long-range electric fields that interact with charge carriers. To validate this mechanism in Mg_3_(Sb, Bi)_2_, we performed first-principles calculations of the phonon dispersion and DOS. As shown in Fig. 2f, distinct longitudinal–transverse optical (LO–TO) phonon splitting is observed near the Γ point of the Brillouin zone, with the LO mode exhibiting a higher frequency than the TO mode. Although substitution of Sb may slightly modify the phonon dispersion, the LO–TO phonon splitting is not expected to be substantially altered [40]. This splitting arises from the additional restoring force induced by lattice polarization [39]. The polar-covalent [Mg_2_Sb_2_]^2−^ units in Mg_3_(Sb, Bi)_2_, arising from the mixed ionic–covalent nature of Mg–Sb bonds, are the primary source of lattice polarization [41]. The substitution of Sb by Sn reduces the electronegativity difference relative to Mg, thereby lowering the bond polarity and enhancing the covalent nature of the Mg–X bonds. Consequently, carrier transport is strongly affected through Fröhlich-type electron–phonon coupling [42], in which LO phonons scatter electrons via the long-range coulombic electric field.

The strength of this coupling is quantified by the dimensionless polar coupling constant *α*_PO_ [18]:


(1) \begin{eqnarray*}
{\alpha }_{{\mathrm{PO}}} = \frac{{{e}^2}}{{4\pi \hbar }}{\left( {\frac{{{m}^{\mathrm{*}}}}{{2\hbar {\omega }_{{\mathrm{LO}}}}}} \right)}^{1/2}\left( {\frac{1}{{{\varepsilon }_\infty }} - \frac{1}{{{\varepsilon }_{\mathrm{s}}}}} \right),
\end{eqnarray*}

where $\hbar$ is the Planck constant, *ω*_LO_ is the LO phonon frequency, and *ɛ*_∞_ and *ɛ*_s_ are high-frequency and static dielectric constants, respectively. As shown in Fig. 2g, typical semiconductors such as Si, Ge and InSb exhibit low *α*_PO_ and monotonic *n–μ* trends, whereas materials such as PbTe/Se/S, half-Heuslers and Zintl compounds show higher *α*_PO_ and suppressed *μ* at low *n*. Notably, Mg_3_Sb_2_ exhibits an even larger *α*_PO_ (Table S3), reinforcing the relevance of polar-optical-phonon scattering in this system. Although polar-optical-phonon scattering is strong at low *n*, the interaction can be screened by increased free carriers. According to the Thomas–Fermi theory [39], screening length is inversely related to *n*, meaning that higher *n* reduces the effective LO–TO splitting by screening long-range Coulomb interactions. Thus, the observed *μ*_w_ suppression in the *x* = 0.04 and *x* = 0.04-T20 samples is attributed to enhanced polar-optical-phonon scattering at lower *n*. The slightly positive temperature dependence of *μ*_w_ indicates the presence of grain-boundary scattering in these samples. At room temperature and above, polar-optical-phonon scattering can be approximately treated as an elastic process, with the relaxation time *τ*_po_ ∝ m*^−1/2^  *T*^−1/2^  *ε*^−1/2^ [22], where *T* is the temperature and *ε* is the carrier energy. Because of its weaker dependence on *T* and m* compared with acoustic-phonon scattering, the contribution of polar-optical-phonon scattering diminishes with increasing temperature and, at high temperature, the transport is dominated by acoustic-phonon scattering, consistently with the observed *T*^−1.5^ behavior of *μ*_w_. Therefore, polar-optical-phonon scattering is mainly relevant in the intermediate temperature range in our Mg_3_(Sb, Bi)_2_ samples and contributes to the averaged *zT*. Furthermore, based on the *n*–*μ* diagram (Fig. 1c), polar-optical-phonon scattering is significant only when *n* < 10^19^ cm^−3^, which accounts for the suppressed *μ* observed in the *x* = 0.04-T20 sample. In our optimized samples, *n* is close to this threshold, but the impact of polar-optical-phonon scattering remains limited.

By balancing all scattering mechanisms—acoustic-phonon, defects, grain-boundary and polar-optical-phonon—we achieved optimized carrier transport. As shown in Fig. 2h, *μ* reaches a maximum of 179 cm^2^ V^−1^ s^−1^ in the *x* = 0.02-T20 sample, representing a 136% enhancement over the undoped reference (76 cm^2^ V^−1^ s^−1^). Further increases in the Sn content, however, trigger enhanced polar-optical-phonon scattering, thereby reducing *μ*. The improved *μ* results in a significantly enhanced *PF*, especially near room temperature (Fig. S7). The SPB model simulations confirm that enhanced *μ* can sustain high *PF* even at moderately reduced *n* (Fig. 2i), underscoring the critical role of *μ* in TE optimization.

### High *zT* enabled by synergistic control of ***μ*** and ***κ***_lat_

**Figure 3.**
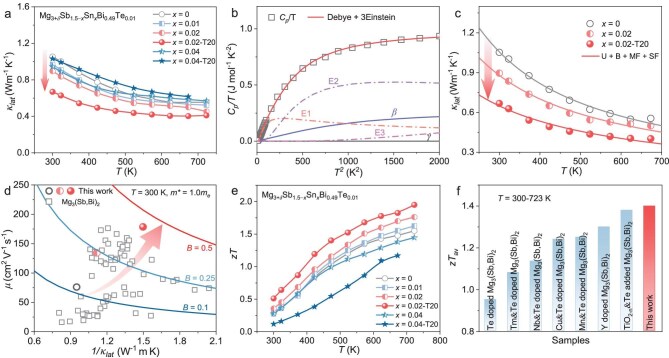
High *zT* enabled by synergistic control of phonon transport and *B* factor. (a) Temperature-dependent *κ*_lat_ of the samples. (b) Plots of *C_p_*/*T* versus *T*^2^ for *x* = 0.02-T20 sample fitted using Debye–Einstein model. (c) Experimental and fitted *κ*_lat_ based on Debye–Callaway model. (d) *μ* versus 1*/κ*_lat_ plots at 300 K (Table S6). Solid lines indicate *B* factor contours calculated with a fixed m* = 1.0 *m*_e_, serving as a general reference for comparison between samples. (e) Temperature-dependent *zT* of the samples. (f) Comparison of the average *zT* (300–723 K) for Mg_3_(Sb, Bi)_2_-based materials (Table S7).

To complement the carrier-transport analysis and demonstrate the comprehensive benefits of *n*–*μ* map-guided optimization, we next examine the thermal transport behavior. As shown in Fig. S8, *κ* decreases after Sn doping and synthesis optimization. To decouple the contributions, *κ*_lat_ was estimated by subtracting the electronic component (*κ*_ele_) from *κ*. As shown in Fig. 3a, *κ*_lat_ systematically decreases with increasing Sn doping and extended sintering time across the entire temperature range. For example, at room temperature, *κ*_lat_ is reduced from 1.05 W m^−1^ K^−1^ in the pristine sample to 0.89 W m^−1^ K^−1^ for *x* = 0.02 and further to 0.67 W m^−1^ K^−1^ for *x* = 0.02-T20. A minimum *κ*_lat_ of 0.40 W m^−1^ K^−1^ is achieved at 673 K in the *x* = 0.02-T20 sample. When the Sn content reaches *x* = 0.04, a small fraction of metallic Sn may precipitate due to the solubility limit. As metallic Sn possesses a much higher *κ* than does the Mg_3_(Sb, Bi)_2_ matrix, its presence, even in small amounts, partially counteracts the phonon-scattering effect and leads to the observed increase in *κ*_lat_.

To elucidate the origin of the reduced *κ*_lat_, low-temperature heat capacity (*C_p_*) measurements were performed. The data for *C_p_*/*T* versus *T*^2^ were well fitted by using the Debye–Einstein model (Fig. 3b and Fig. S9) [44]:


(2) \begin{eqnarray*}
\frac{{{C}_p}}{T} &=& \\gtrsimmma + \beta {T}^2\nonumber\\
&&+\, \mathop \sum \limits_{i = 1}^n \left({A}_i\Theta _{Ei}^2{({T}^2)}^{ - \frac{3}{2}}\frac{{{e}^{\frac{{{\Theta }_{Ei}}}{T}}}}{{{{({e}^{\frac{{{\Theta }_{Ei}}}{T}} - 1)}}^2}}\right)\!,
\end{eqnarray*}

where *γ* is the Sommerfeld coefficient representing the electronic contribution, the second term stands for the Debye lattice component and the final term accounts for localized Einstein oscillators, with *A_i_* as the prefactor of each Einstein mode. The fitted Debye temperatures (*Θ*_D_) are 219 and 216 K for the *x* = 0 and *x* = 0.02-T20 samples (Table S4), suggesting lattice softening after structural modulation. In addition, a Boson-like feature is observed in the plot of *C_p_*/*T*^3^ versus *T* at low temperatures (Fig. S9) [45], indicating deviations from the conventional Debye law. This feature is attributed to excess phonon DOS originating from low-energy optical phonons, which hinder heat transport by scattering long-wavelength acoustic phonons. Consistently, a reduction in the lowest Einstein temperature (*Θ*_E1_) is observed in the *x* = 0.02-T20 sample, reinforcing the role of low-frequency optical modes in *κ*_lat_ suppression.

To further quantify the phonon-scattering contributions, we employed the Debye–Callaway model to fit the experimental *κ*_lat_ data [46]. The fitting accounts for multiple scattering sources, including Umklapp processes (U), grain boundaries (B), mass fluctuation (MF) and strain fluctuation (SF), with the parameters summarized in Table S5. As shown in Fig. 3c, the reduction in *κ*_lat_ primarily arises from enhanced MF and SF scattering, which can be associated with point defects and lattice disorder introduced by Sn substitution and Mg loss during sintering. Furthermore, the strengthened phonon–phonon U scattering aligns with the *C_p_* analysis. Together, these results demonstrate that the structural modifications introduced to optimize the carrier transport also concurrently intensify the phonon scattering, leading to suppressed *κ*_lat_ and improved TE performance.

Owing to the concurrent enhancement of *μ* and suppression of *κ*_lat_, a significant increase in the *B* factor is observed. As shown in Fig. 3d, a comparative plot of *μ* versus 1*/κ*_lat_ across the various Mg_3_(Sb, Bi)_2_ compositions reveals a clear upward shift for the *x* = 0.02-T20 sample. The contour lines represent iso-*B* values assuming a constant m*, highlighting the remarkable increase in the *μ/κ*_lat_ ratio achieved in this work. Notably, the *B* factor exceeds 1.0 at 723 K (Fig. S8), indicating a highly favorable transport balance. As a direct outcome, *zT* is substantially improved across the entire temperature range. The *x* = 0.02-T20 sample exhibits a *zT* of ∼0.5 at room temperature and peak *zT* of ∼2.0 at 723 K (Fig. 3e). More significantly, the average *zT* over 300–723 K exceeds 1.4 (Fig. 3f), surpassing all previously reported values for Mg_3_(Sb, Bi)_2_-based materials. The excellent reproducibility of these results is confirmed by using independent measurements on multiple batches (Fig. S10), underscoring the robustness of the optimization strategy. Importantly, these results confirm that our *n*–*μ* map-guided doping and processing strategy not only improves carrier transport, but also tailors phonon-scattering pathways, thereby synergistically boosting *zT* and laying a solid foundation for high-efficiency devices.

### High-performance Mg-based TE modules for efficient power generation

To evaluate the real-world energy-conversion performance of our optimized Mg_3_(Sb, Bi)_2_ materials, particularly in view of their high average *zT* and *μ*–*κ*_lat_ balance, we fabricated a single-leg TE module. Mg was employed as the interfacial material, yielding an ultralow contact resistivity of 1.8 μΩ cm^2^ in the Mg/Mg_3_(Sb, Bi)_2_ joints, which remained stable after annealing at 593 K for 7 days (Fig. S11). SEM and EDS confirmed a well-defined interface structure containing an ∼10-μm Mg interlayer after aging, effectively suppressing Mg loss into the contact layer and thereby guaranteeing dual stability in both contact resistivity and material performance (Fig. S12) [47].

**Figure 4.**
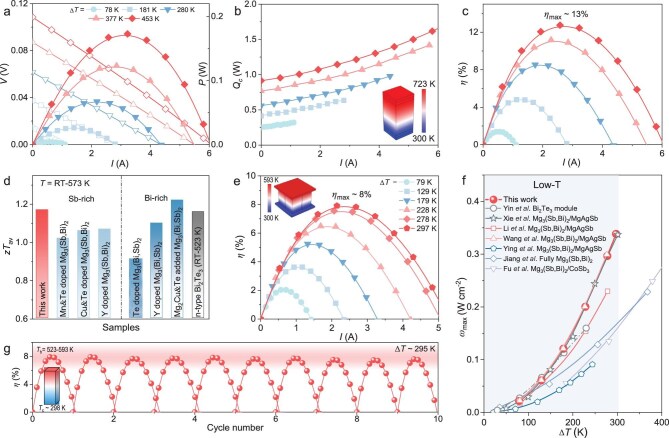
Superior performance of Mg-based TE modules. *I*-dependent performance of the single-leg module under various Δ*T*: (a) *V* and *P*, (b) *Q*_c_ and (c) *η*. (d) Comparison of the average *zT* (300–573 K) with those of previously reported Mg_3_(Sb, Bi)_2_ samples and n-type Bi_2_Te_3_ (Table S7). (e) *η* as a function of *I* under different Δ*T*; inset shows the design of the two-pair module. (f) Comparison of *ɷ*_max_ of the two-pair Mg-based module with the literature [31,32,34,35,37,53,54]. (g) Thermal cycling performance of the two-pair Mg-based module.

The measured output voltage (*V*), output power (*P*), heat flow from the cold side (*Q*_c_) and conversion efficiency (*η*) are shown in Fig. 4a–c. A maximum *P* of 0.17 W was achieved under a temperature gradient (Δ*T*) of 453 K, corresponding to a maximum power density (*ɷ*_max_) of 1.24 W cm^−2^. The single-leg module exhibited a maximum conversion efficiency (*η*_max_) of ∼13% at Δ*T* = 453 K. Theoretical simulations indicate that *η*_max_ could reach 17.2% (Fig. S13), suggesting that the experimental–theoretical gap is mainly attributed to interfacial contact resistance and uncertainties in the heat-flow measurement. Despite these factors, both the experimental and simulated results demonstrate the strong potential of Mg_3_(Sb, Bi)_2_ for efficient, low-cost TE power generation.

Given that much of the waste heat in real-world applications exists in the low-grade temperature range, we further explored the feasibility of constructing an all-Mg-based module by pairing Mg_3_(Sb, Bi)_2_ with p-type MgAgSb. As shown in Fig. 4d, our optimized Mg_3_(Sb, Bi)_2_ sample exhibits a significantly higher average *zT* than in previous reports and outperforms n-type Bi_2_Te_3_ within the relevant temperature window. Although Bi-rich Mg_3_(Sb, Bi)_2_ alloys can exhibit comparable *zT* values at low temperature due to their higher *μ* from reduced grain-boundary scattering and lower m* [48], their relatively lower chemical and thermal robustness may limit long-term deployment (Fig. S14) [49, 50].

To overcome these limitations, we designed and fabricated a two-pair TE module composed entirely of Mg-based materials. The resulting device, using our high-performance n-type Mg_3_(Sb, Bi)_2_ and p-type MgAgSb [51], offers a scalable, low-cost alternative to conventional Bi_2_Te_3_ systems for low-grade heat recovery. Detailed output characteristics—including current (*I*)-dependent *V, P* and *Q*_c_—are shown in Fig. S15. The module achieves *η*_max_ of ∼8% under a Δ*T* of 297 K (Fig. 4e). Due to challenges in accurately measuring the heat flow [52], especially at elevated temperatures, the experimental *Q*_c_ values tended to exceed simulated predictions (Figs S16 and S17). Using simulated *Q*_c_ and measured *P*, the recalculated *η*_max_ reaches 9.3% (Fig. 1f), confirming the module’s strong potential for applications at <300 K. Simulation results further suggest that *η*_max_ could reach 12.1% under the same conditions (Fig. S17), showcasing the potential for further improvement. Moreover, the module delivers a *ɷ*_max_ of 0.34 W cm^−2^ (Fig. 4f), outperforming both Bi_2_Te_3_-based and previously reported Mg_3_(Sb, Bi)_2_-based modules [31, 32, 34, 35, 37, 53, 54]. A significant resistance gap exists at the junctions between the metallization and the electrode layer (Fig. S11), which can partially limit the effective *I* and thus reduce the *P*. Such a high *ɷ*, driven by an enhanced *PF*, is particularly advantageous for space-constrained applications, such as wearable electronics or TE-powered Internet of Things (IoT) sensors. Finally, thermal cycling tests—performed by repeatedly heating and cooling the device—showed negligible degradation in *η*_max_ (∼8%) after 10 cycles (Fig. 4g), suggesting the good initial thermal durability of our Mg-based system under low-grade heat conditions. To further assess the long-term stability of Mg-based modules, other factors such as soldering and chemical stability should be carefully considered in future work.

## CONCLUSION

In summary, we constructed a *n*–*μ* diagram for Mg_3_(Sb, Bi)_2_ by systematically identifying the carrier-scattering mechanisms—including acoustic-phonon, grain-boundary, defect and polar-optical-phonon scattering. Guided by this framework, we implemented a targeted doping–processing strategy that enabled an ultra-high *μ* of 179 cm^2^ V^−1^ s^−1^, while concurrently suppressing phonon transport through microstructural engineering. This dual optimization yielded a peak *zT* of ∼2.0 and an average *zT* of 1.4 over 300–723 K. Device-level evaluations confirmed the high performance, with single-leg modules achieving *η*_max_ of ∼13% and fully Mg-based modules realizing ∼8% efficiency, underscoring the potential for scalable waste-heat harvesting. Beyond this promising material system, our study offers an applicable approach for transport optimization in complex TE semiconductors.

## METHODS

### Materials synthesis and module fabrication

To synthesize the Mg_3.2+2_*_x_*Sb_1.5–_*_x_*Sn*_x_*Bi_0.49_Te_0.01_ compounds (*x* = 0, 0.01, 0.02, 0.04, referred to as Mg_3+_*_δ_*Sb_1.5–_*_x_*Sn*_x_*Bi_0.49_Te_0.01_ in the main text), raw materials of Mg (99.95%), Te (99.999%), Sb (99.999%), Bi (99.999%) and Sn (99.99%) were stoichiometrically weighed inside an argon-filled glovebox and loaded into a stainless-steel ball-milling jar. The samples underwent a high-energy ball-milling process for 5 hours (SPEX 8000D). The resulting powders were consolidated by using spark plasma sintering (SPS, SPS-1080 System, SPS SYNTEX INC) under a pressure of 60 MPa at 973 K for 10–20 minutes. Here, the *x*-T20 samples denote a doping level of *x* and a holding time of 20 min. The p-type MgAgSb samples were synthesized by ball milling with 0.625 wt% of C_18_H_36_O_2_ additive and subsequently densified by using SPS at 573 K under 60 MPa for 5 minutes (SPS-322Lx, Dr. Sintering).

Single-leg modules were fabricated by assembling Ni/Mg/Mg_3_(Bi, Sb)_2_/Mg/Ni junctions via the SPS process and the details can be found in our previous work [47]. The sintered sandwich structures were ground, polished and diced into ∼3.8 mm × 3.8 mm × 6 mm pieces for performance measurement. For the p-type legs, an Sb-based alloy was used as the contact layer and the legs were fabricated via a single-step SPS sintering process. Two-pair modules were fabricated based on these n-type and p-type legs. Module electrical output power and generation performance were measured by using commercial apparatus (Mini-PEM, ADVANCE RIKO, Japan). The hot-side temperature (*T*_h_) was controlled by using a heater and the cold-side temperature (*T*_c_) was maintained via a water flow. The output power (*P*) and cold-side heat flow (*Q*_c_) were measured by using Mini-PEM, so the energy-conversion efficiency was calculated by using the equation *η* = *P*/(*P* + *Q*_c_). The power density *ɷ* was calculated by using the standard formula *ɷ* = *P*/*A*, where *A* is the cross-sectional area of the module (1.0 cm^2^). Thermal-cycle measurements were performed with a fixed *T*_c_ of ∼298 K and *T*_h_ fluctuating between 523 and 593 K. Three-dimensional finite-element simulations of power generation were conducted using COMSOL Multiphysics^®^ by coupling the Heat Transfer in Solids, Electric Currents and Thermoelectric Effect interfaces to capture the coupled thermal and electrical transport in the devices. Thermal and electrical contact resistances at interfaces were neglected in the simulation.

## Supplementary Material

nwaf507_Supplemental_File
