# Supplementary material for: Leveraging carrier mobility enables high-performance Mg3(Sb, Bi)2 thermoelectrics
Source: Natl Sci Rev. 2025 Nov 17;13(1):nwaf507. doi: 10.1093/nsr/nwaf507 (PMC12796818; doi:10.1093/nsr/nwaf507)
Supplement: nwaf507_Supplemental_File [file nwaf507_supplemental_file.pdf]

Supplementary data

Leveraging carrier mobility enables high-performance $Mg_3(Sb,Bi)_2$ thermoelectrics

Longquan Wang[1,2,†], *Airan Li*[1,†], *Xinzhi Wu*[1†], *Jiankang Li*[1,2], *and Takao Mori*[1,2]*

[1] Research Center for Materials Nanoarchitectonics (MANA), National Institute for Materials Science (NIMS), Namiki 1-1, Tsukuba 305-0044, Japan

[2] Graduate School of Pure and Applied Sciences, University of Tsukuba, Tennodai 1-1-1, Tsukuba 305-8671, Japan

*Corresponding author, e-mail: MORI.Takao@nims.go.jp
†These authors contribute equally to this work

SUPPLEMENT METHODS

Properties and microstructure characterization

The electrical properties, including σ and S, were measured under helium atmosphere using a ZEM-2 system (ADVANCE RIKO, ± 5 % uncertainty). The κ was calculated as $\kappa = \lambda C_p d$, where thermal diffusivity λ was obtained by a laser flash method (LFA 467, NETZSCH, ± 3 % uncertainty), C_p is the heat capacity, and d is the density measured by an Archimedes method. The C_p was calculated using the polynomial equation from Agne et al.[1]: $C_p = 3NR\ (1 + 1.3 \times 10^{-4}\, T - 4 \times 10^{3}\, T^{-2}) / M_w$, where N is the number of atoms per formula unit and M_w is the molecular weight. Microstructure and composition analyses were characterized via a field-emission scanning electron microscope (FESEM, Hitachi SU8000) with energy dispersive spectrometer (EDS, XFlash FlatQUAD 5060F). Phase identification employed X-ray diffractometer (SmartLab3, Rigaku) with Cu Kα radiation operating at 40 kV × 15 mA, scanning at 3°/min. Hall n was measured using a Physical Property Measurement System (PPMS, Quantum Design) equipped with an AC transport option, while low-temperature C□ was measured using the heat capacity option. Contact resistance at room temperature was determined with a two-axis resistance distribution instrument (S1331, Mottainai energy).

Theoretical calculations

Phonon dispersion relations and phonon DOS were computed within the harmonic approximation using the finite displacement method as implemented in Phonopy[2]. Density functional theory (DFT) calculations were performed using the Vienna Ab initio Simulation Package (VASP) with the projector augmented wave (PAW) method and the Perdew-Burke-Ernzerhof (PBE) form of the generalized gradient approximation (GGA)[3]. A 2×2×2 supercell of the conventional cell was used with a Γ-centered k-point mesh.

Debye-Callaway model

In this work, the lattice thermal conductivity was analyzed using the Debye-Callaway model, which provides a quantitative framework for assessing multiple phonon scattering processes. The model is expressed as[4]:

$$\kappa_L(T) = \frac{k_B}{2\pi^2 v_s}\left(\frac{k_B T}{\hbar}\right)^3 \int_0^{\frac{\theta_D}{T}} \frac{x^4 e^x}{\tau_{ph}^{-1}(e^x - 1)^2} dx \#(S1)$$

Where $x = \hbar\omega/k_B T$, and v_s is the average sound velocity. The phonon relaxation time τ_{ph}^{-1} accounts for several scattering mechanisms:

$$\tau_{ph}^{-1} = \frac{V}{4\pi v_s^3}(\Gamma_{MF} + \Gamma_{SF})\omega^4 + \tau_U^{-1} + \frac{v_s}{L} \#(S2)$$

The first term captures defects scattering contributions from mass and strain field fluctuations, characterized by Γ_{MF} and Γ_{SF}, respectively. The second term describes Umklapp scattering:

$$\tau_U^{-1} = U\omega^2 T^n \exp\left(\frac{-\theta_D}{mT}\right) \#(S3)$$

where U is estimated from material parameters as:

$$U = \frac{\hbar\gamma^2}{M v_s^2 \theta_D} \#(S4)$$

where γ is the Gruneisen parameter, M is the average atomic mass, and θ_D is the Debye temperature. Experimentally determined values from sound velocity (Supplementary Table 7), grain size, and low-temperature heat capacity were used in the fitting.

The scattering term Γ due to mass fluctuation can be calculated using the chemical composition and the expression is given by Gurunathan et al. for multi-component alloy[5]:

$$\Gamma_{MF} = \frac{\langle\overline{\Delta M^2}\rangle}{\langle\bar{M}\rangle^2} \#(S5)$$

where the numerator and the denominator can be calculated as:

$$\langle \bar{M} \rangle = \frac{\sum_n c_n \bar{M}_n}{\sum_n c_n}, \quad \bar{M}_n = \sum_i f_{i,n} M_{i,n} \#(S6)$$

$$\langle \overline{\Delta M^2} \rangle = \frac{\sum_n c_n \overline{\Delta M_n^2}}{\sum_n c_n}, \quad \overline{\Delta M_n^2} = \sum_i f_{i,n} (M_{i,n} - \overline{M_n})^2 \#(S7)$$

where *n* is the site index and *i* is the fraction of element at site *n*.

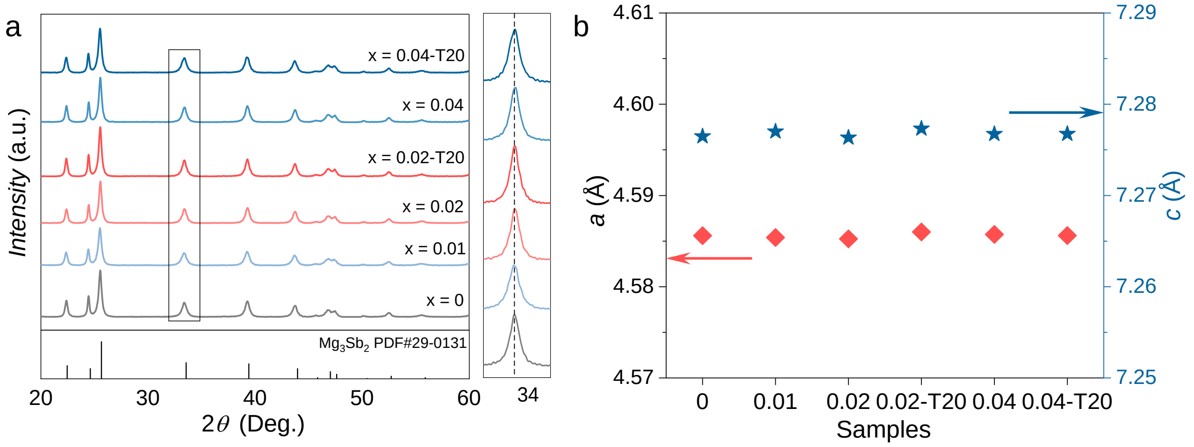

Figure S1. Phase structure. (a) XRD patterns of $Mg_{3+\delta}Sb_{1.5}Sn_xBi_{0.49}Te_{0.01}$ samples and corresponding (b) fitted lattice parameters.

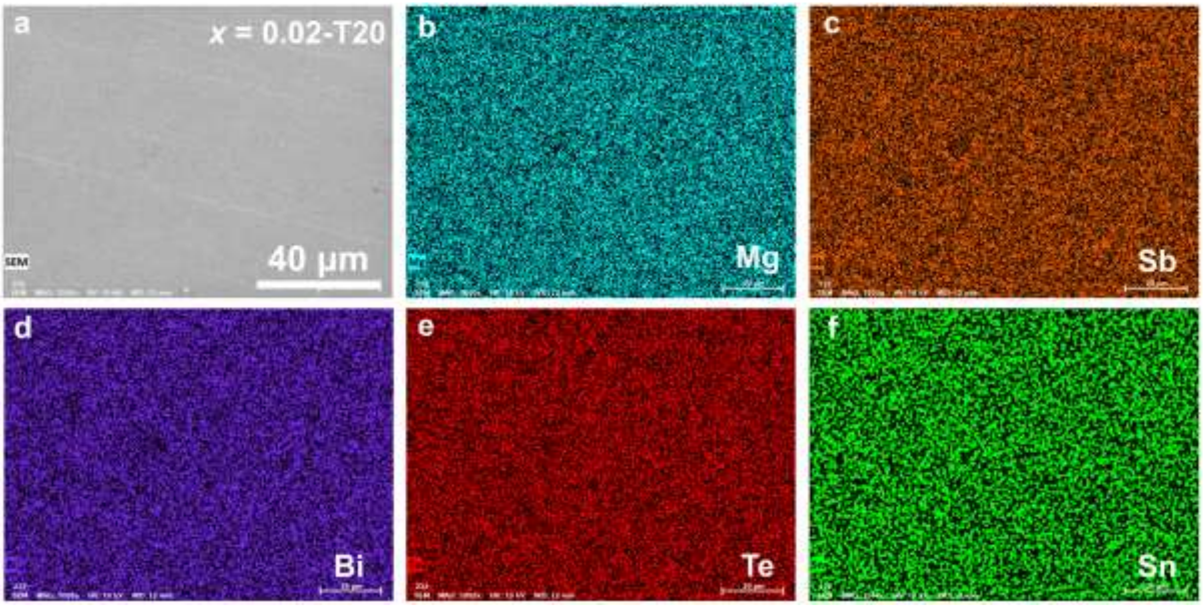

Figure S2. SEM characterization**.** (a–f) SEM and corresponding EDS element mapping of the $x = 0.02$-T20 sample.

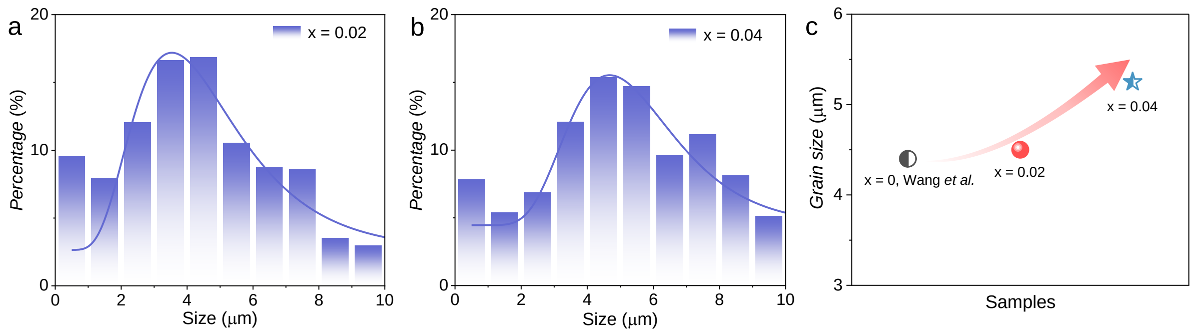

Figure S3. Average grain size. Grain size distribution statistics for (a) $x = 0.02$, (b) $x = 0.04$ samples. (c) The increase in average grain size upon Sn doping[6].

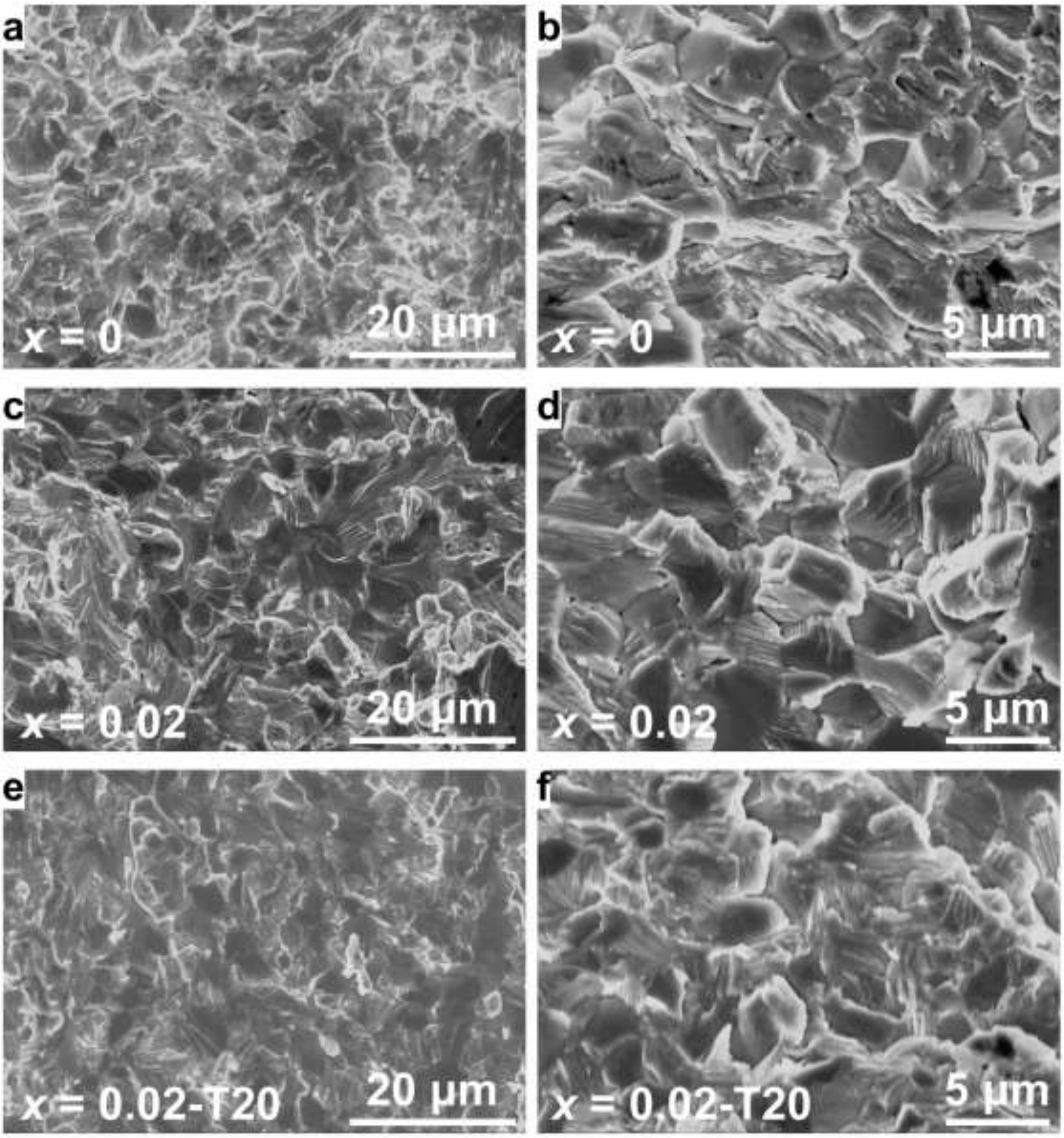

Figure S4. Microstructural characterization by SEM. The SEM images of the fracture morphology for (a–b) $x = 0$, (c–d) $x = 0.02$, (e–f) $x = 0.02$-T20 samples.

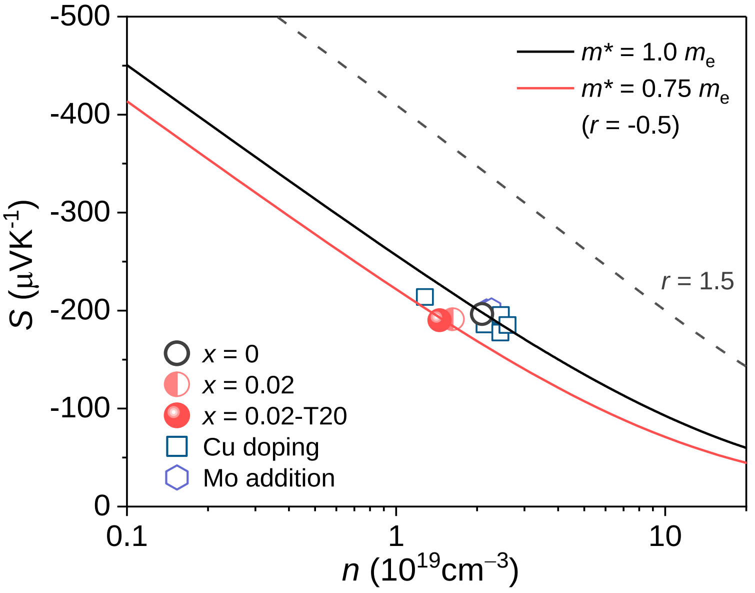

Figure S5. Pisarenko relationship. The S as a function of n at 300 K[7, 8].

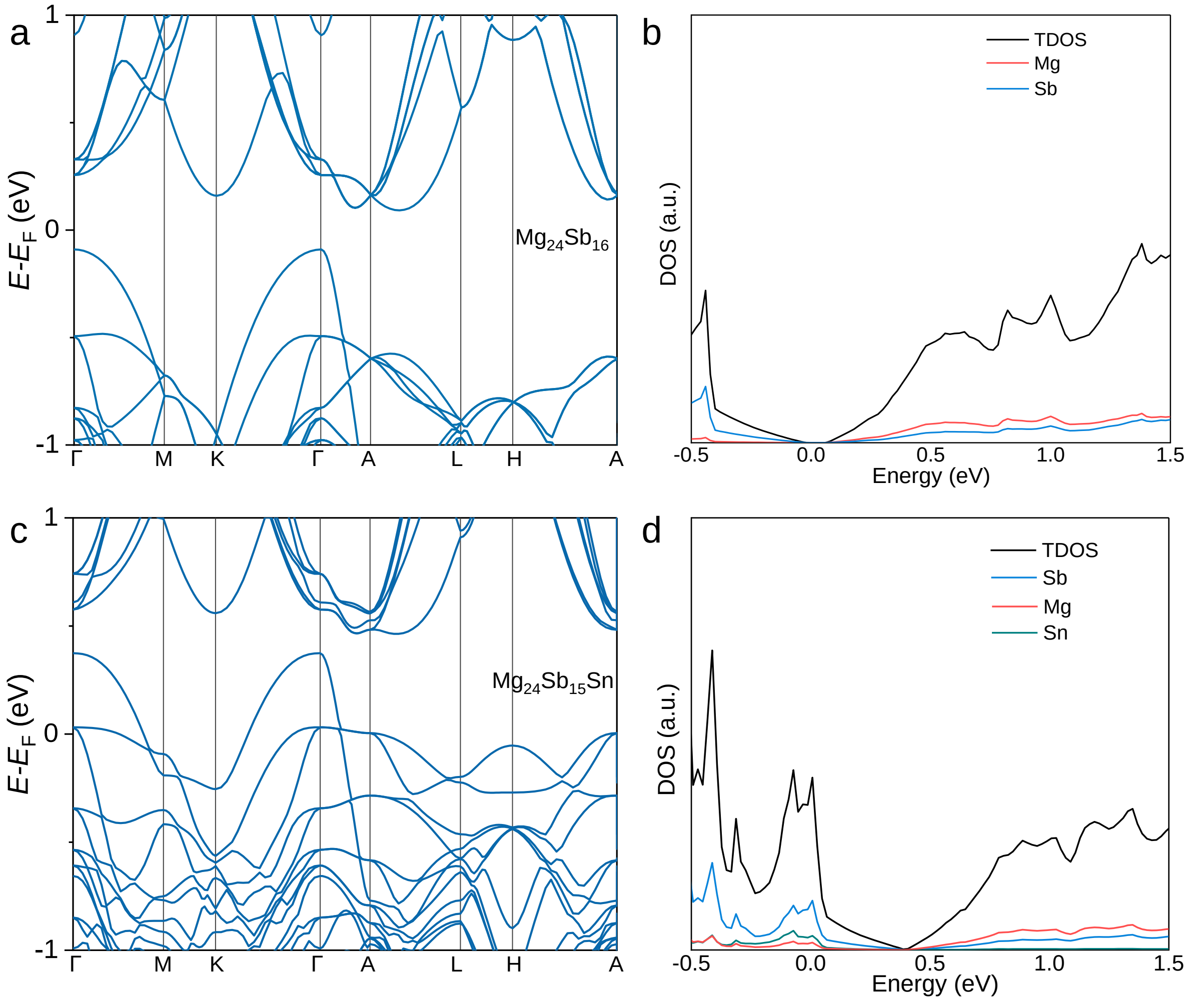

Figure S6. Calculation results. (a) Band structure and (b) partial DOS of pristine Mg_3Sb_2 ($Mg_{24}Sb_{16}$). (c) Band structure and (d) partial DOS of Sn doped Mg_3Sb_2 ($Mg_{24}Sb_{15}Sn$).

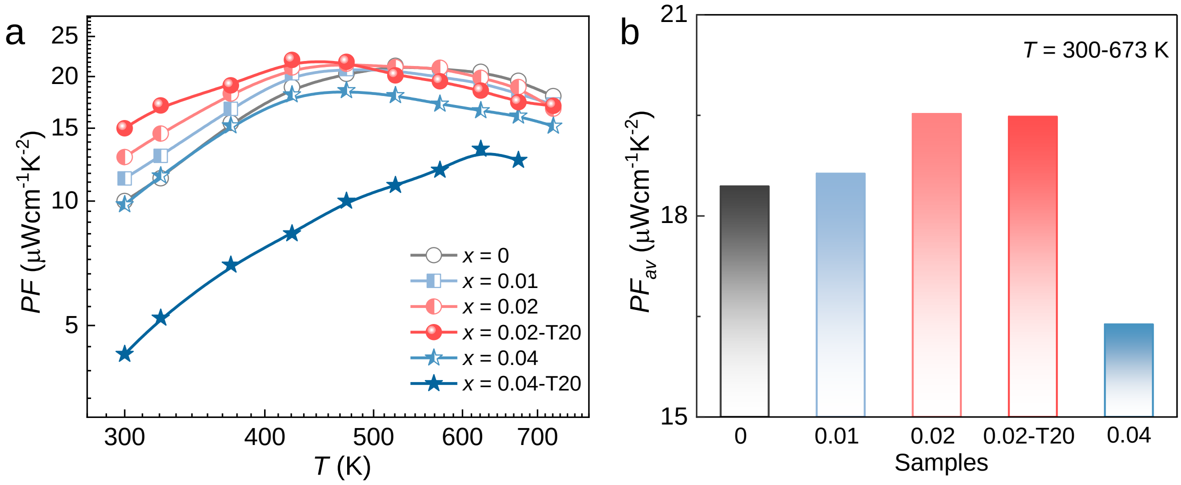

Figure S7. *PF* comparison. (a) Temperature-dependent *PF* of the samples. (b) Comparison of the average *PF* values within the temperature range of 300–673 K.

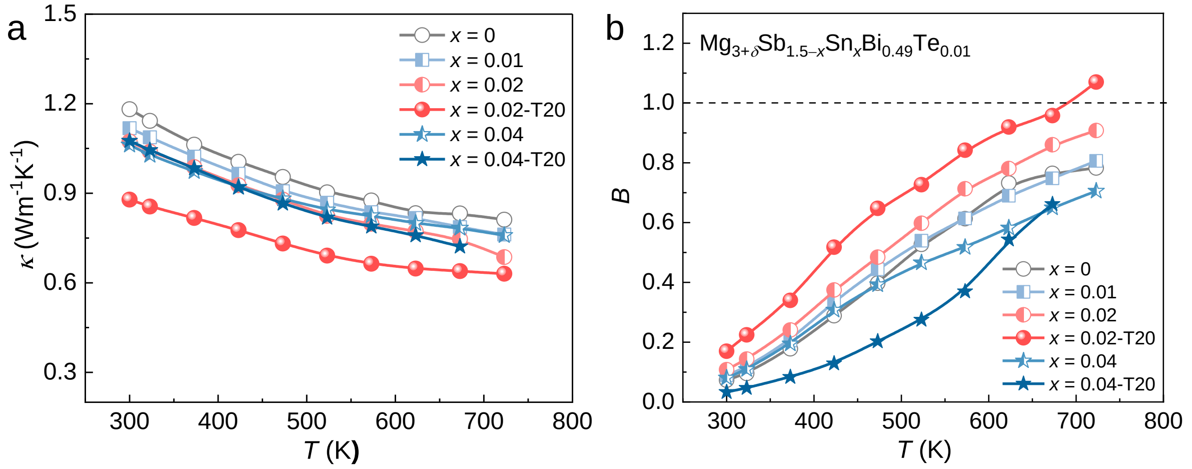

Figure S8. Thermal transport properties and quality factor. Temperature-dependent (a) κ, (b) B factor for the samples.

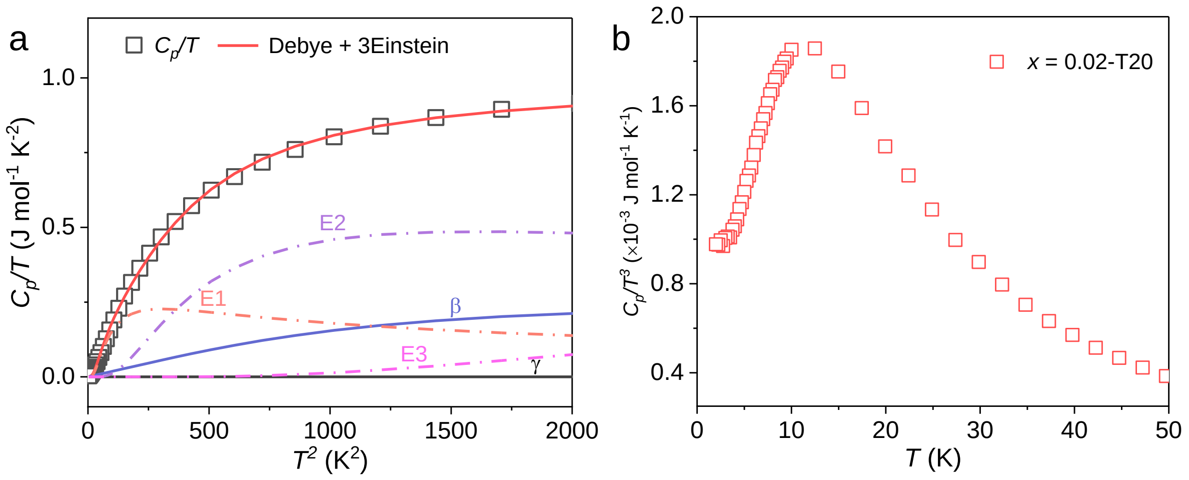

Figure S9. Low-temperature C_p analysis. (a) C_p/T verse T^2 plots for the x = 0 sample. (b) C_p/T^3 verse T^2 plots exhibiting a broad peak between 2–50 K, indicative of the contribution from Einstein modes.

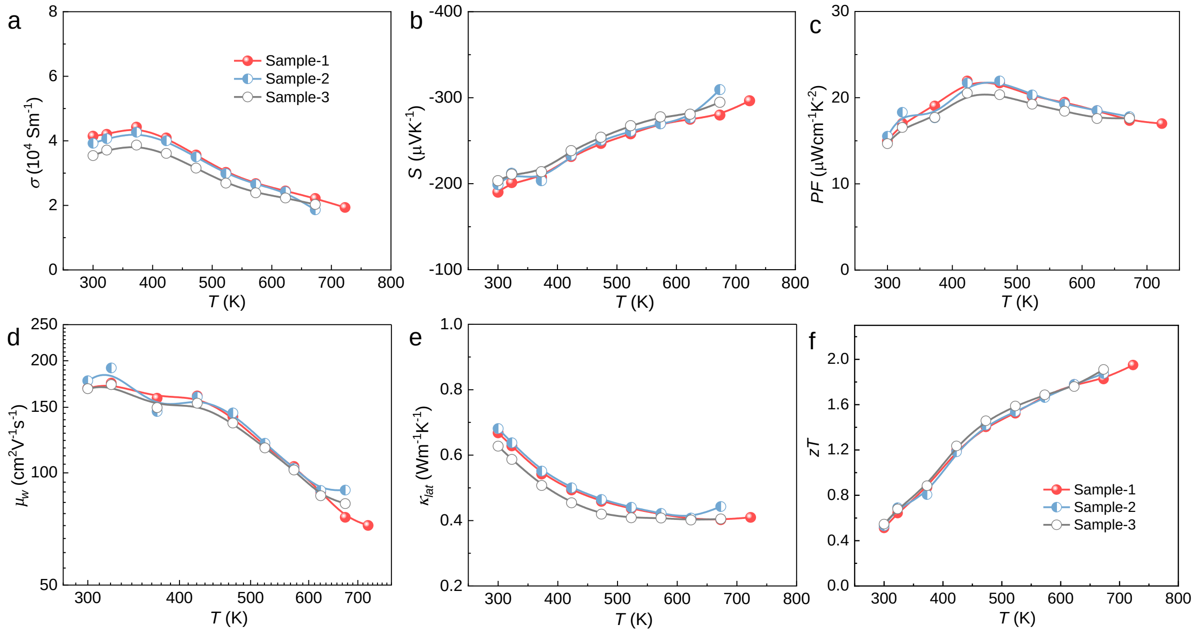

Figure S10. Reproducibility of the TE performance. Temperature-dependent (a) σ, (b) S, (c) PF, (d) μ_{w}, (e) κ_{lat}, (f) zT for the x = 0.02-T20 samples.

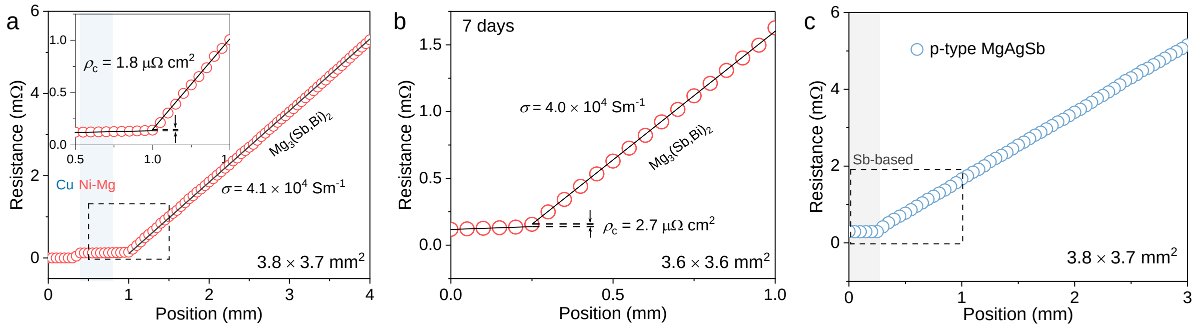

Figure S11. Linear resistance scans. The $Mg/Mg_3(Sb,Bi)_2$ junctions of (a) the as-sintered (0 days) and (b) after aging at 593 K for 7days. (c) The p-type Sb/MgAgSb junctions.

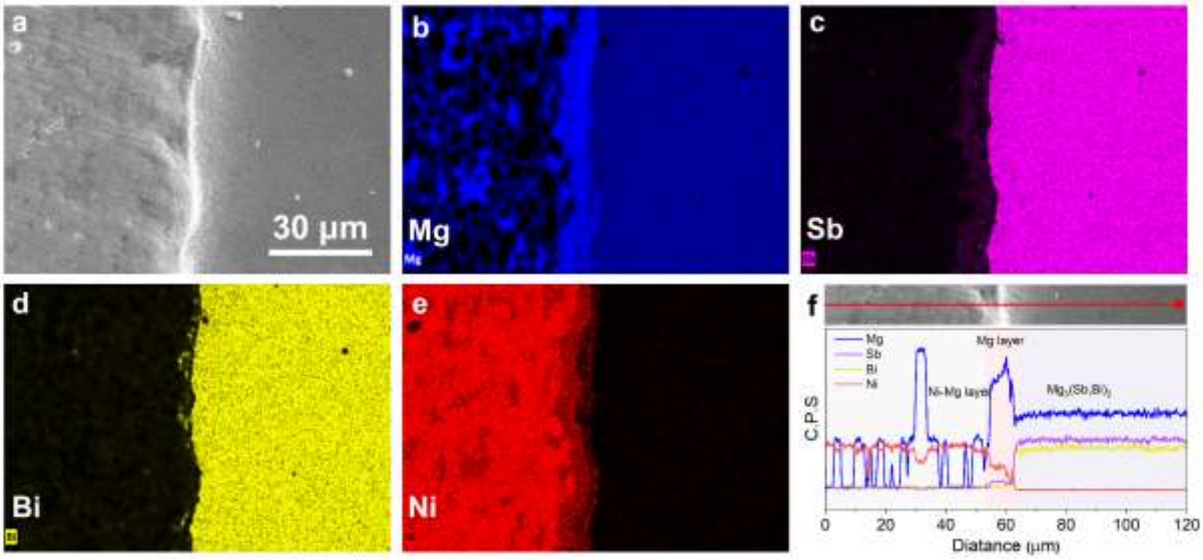

Figure S12. SEM and EDS analysis of the Ni/Mg/$Mg_3(Sb,Bi)_2$ junction after aging. (a) SEM image; EDS mapping for (b) Mg, (c) Sb, (d) Bi and (e) Ni; (f) line scan across the junction.

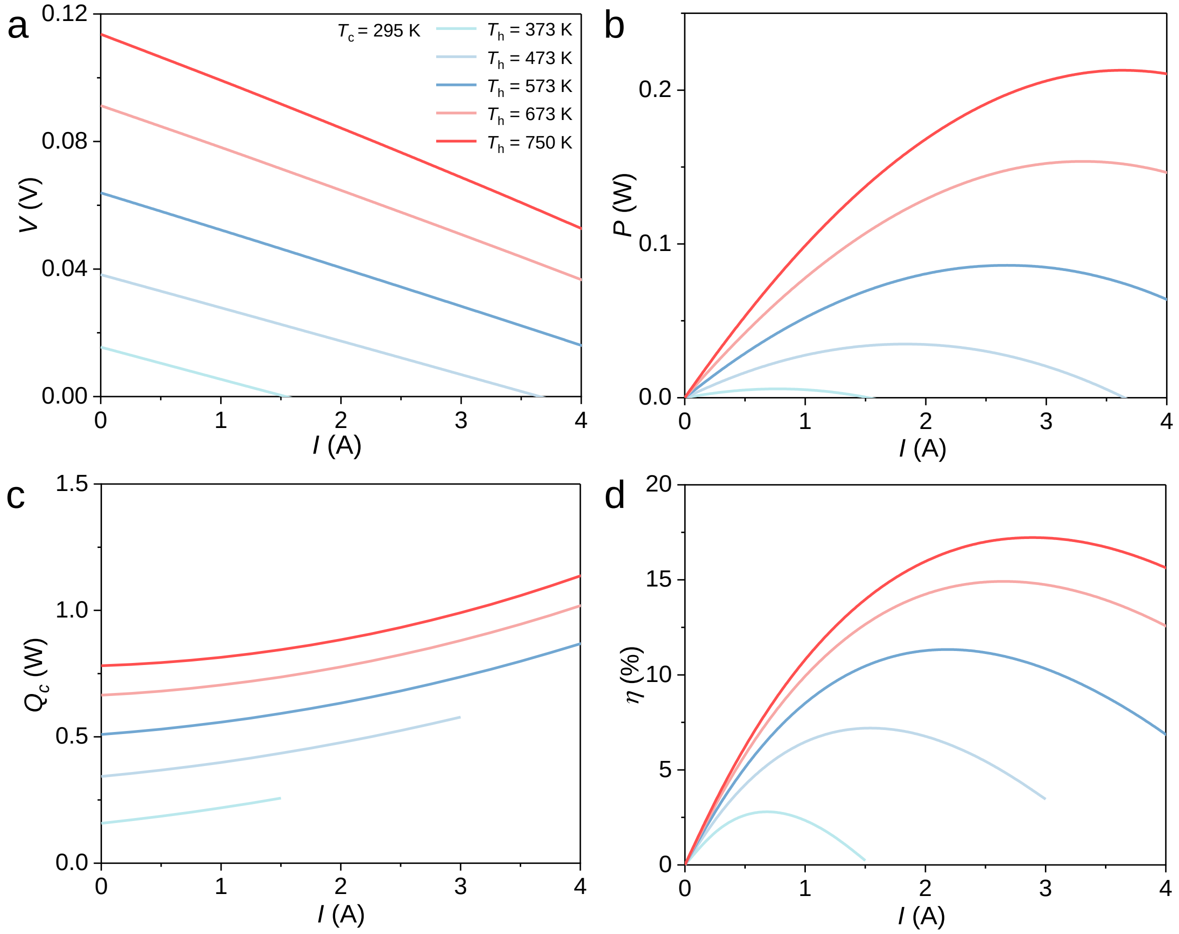

Figure S13. Simulation of the single-leg module. *I*-dependent (a) *V*, (b) *P*, (c) Q_c (d) η of the single-leg module based on optimized $Mg_3(Sb,Bi)_2$ materials.

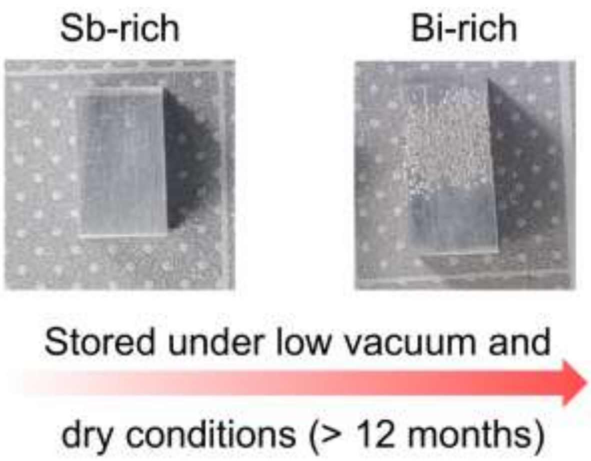

Figure S14. Chemical stability. Optical images of Sb-rich ($Mg_3Sb_{1.5}Bi_{0.5}$) and Bi-rich ($Mg_3Bi_{1.4}Sb_{0.6}$) $Mg_3(Sb,Bi)_2$ samples after storage under low vacuum and dry conditions.

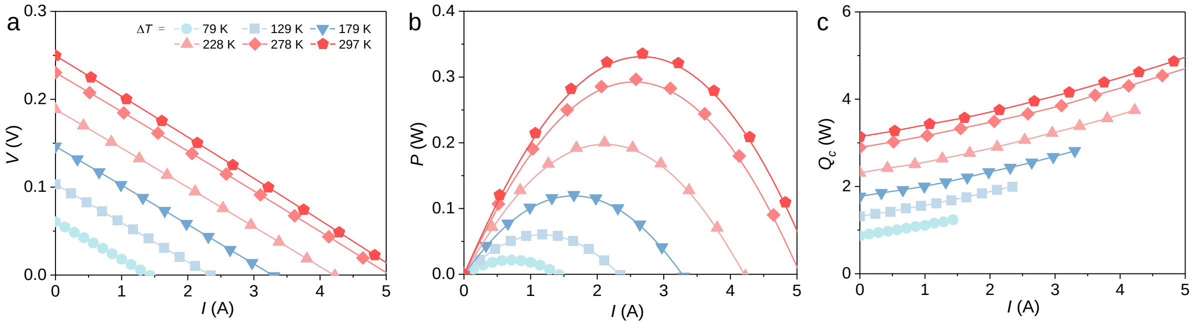

Figure S15. Performance properties of the two-pair module. *I*-dependent (a) *V*, (b) *P*, (c) Q_c of the two-pair module.

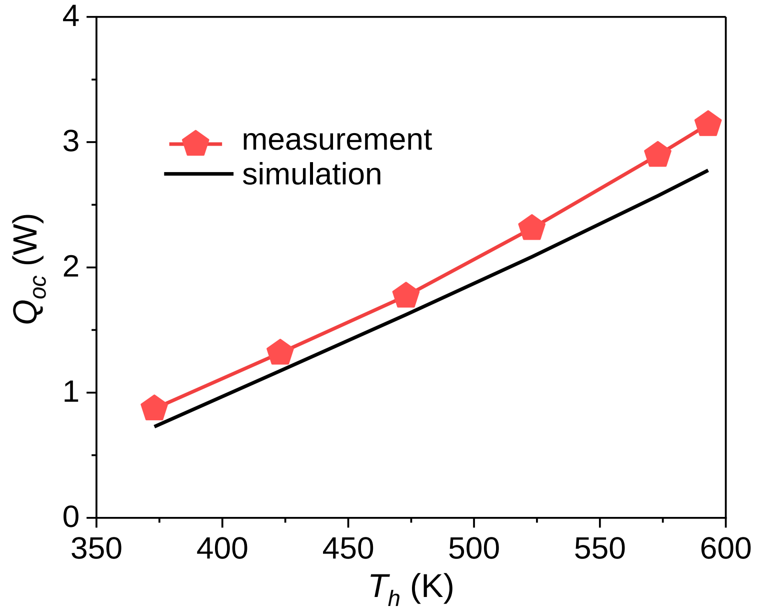

Figure S16. Heat flow. Comparison between simulated and measured Q_c in the two-pair module.

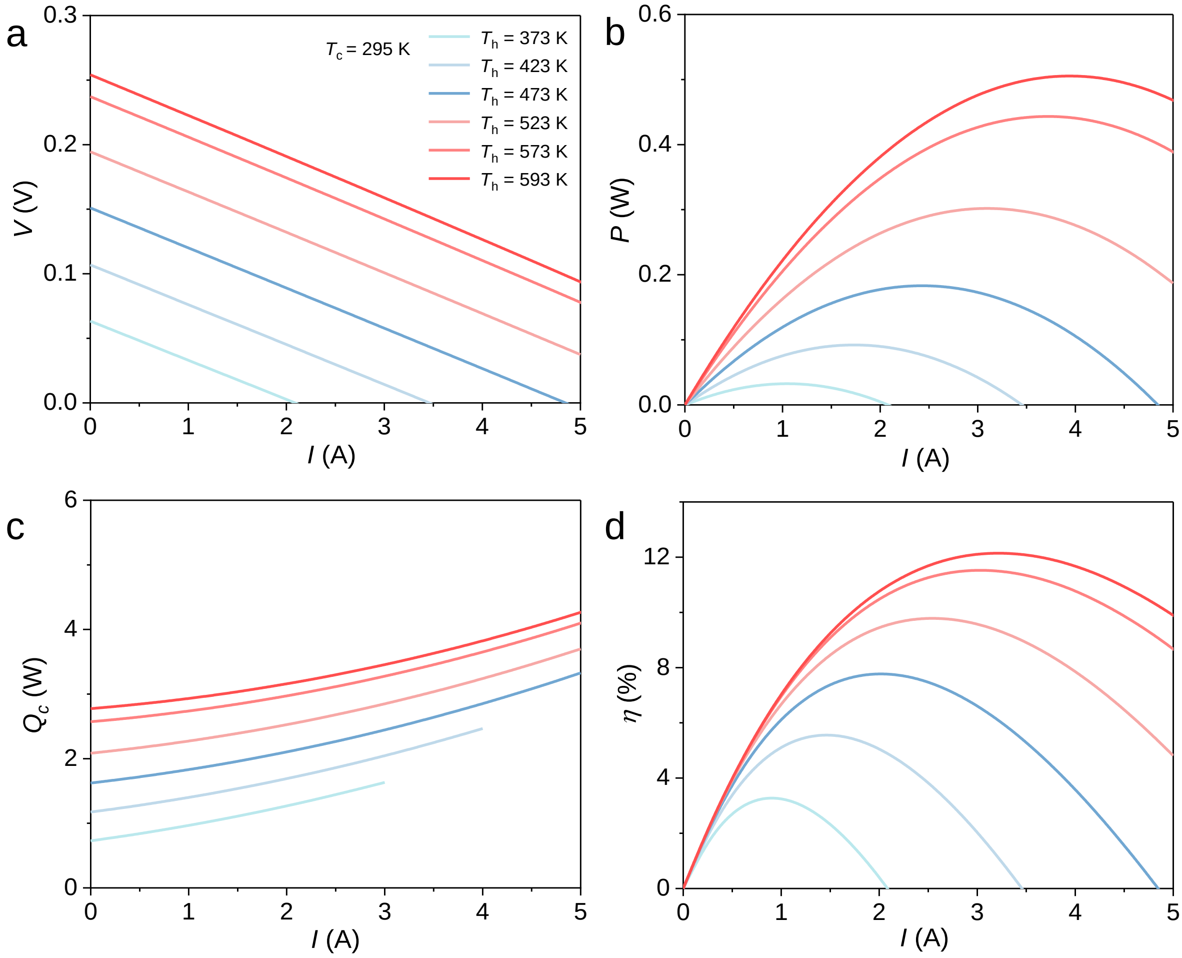

Figure S17. Simulation of the two-pair module. *I*-dependent (a) *V*, (a) *P*, (c) Q_c (d) η of the two-pair module based on optimized $Mg_3(Sb,Bi)_2$ and MgAgSb materials.

Supplementary Table 1. The data of n and μ of $Mg_3(Sb,Bi)_2$-based materials reported in previous studies.

Materials	n ($10^{19}cm^{-3}$)	μ ($cm^2V^{-1}s^{-1}$)	Ref.	Materials	n ($10^{19}cm^{-3}$)	μ $cm^2V^{-1}s^{-1}$)	Ref
Cu&Te codoping	2.45	117.59	Liu *et al.*[7]	S doping	0.39	59.65	Zhang *et al.*[9]
	2.45	134.38			0.58	37.58	
	2.57	149.12			0.57	28.79	
	1.29	173.00			0.50	40.81	
Te doping	3.46	16.48	Shuai *et al.*[10]	Se doping	1.15	67.36	Zhang *et al.*[11]
	4.85	16.89			1.89	42.40	
	5.05	18.12			1.76	32.96	
	5.79	18.88			1.69	48.12	
Mn&Te codoping	3.00	58.19	Chen *et al.*[12]	TM&Te codoping	2.75	124	Jiang *et al.*[13]
	3.20	52.65			2.79	148.88	
	3.30	43.61			2.83	159.76	
	3.11	39.62			3.34	141.36	
TM&Te codoping	3.30	54.12	Mao *et al.*[14]	Ho doping	7.70	16.14	Chen *et al.*[15]
	4.41	61.90			7.91	17.48	
	3.46	72.16			8.26	28.67	
	3.21	74.66					
	3.40	80.60					
Te doping	0.0019	32	Shuai *et al.*[16]	Ti&Te codoping	2.79	155.66	Jiang *et al.*[17]
	1.4	18			2.67	147.26	
	1.8	44			2.54	141.44	
	5.9	19			2.94	144.64	
Gd doping	0.071	107.13	Lei *et al.*[18]	Pr doping	2.21	7.21	Li *et al.*[19]
	2.49	129.46			4.75	10.90	
	3.46	146.88			5.85	20.57	
	4.84	122.60			7.08	18.18	
	5.71	97.73					
	8.93	64.47					
Y doping	1.9	122	Shi *et al.*[20]	In&Te codoping	1.98	91.46	Wang et al.[6]
	2.2	126			1.90	145.92	
	3.5	136			1.81	173.74	
	5.5	125					
	4.3	115					
	6.6	120					
	7.1	120					
	6.1	126					
	8.5	115					
	8.6	95					
Te doping	1.79	92.86	Zhang *et al.*[21]	Se doping	0.91	75.75	Zhang *et al.*[22]
	2.08	81.34			0.73	64.70	
	2.17	62.11			0.70	63.29	
	2.20	44.27			0.80	60.43	
					0.67	62.23	

Supplementary Table 2. Summary of peak zT values of representative TE material systems and the $\eta_{□ax}$ of single-leg modules reported in previous studies.

Samples	zT_{peak}	Ref	Single-leg	η_{max} (%)	Ref
$Mg_2(Si,Sn)$	1.33	Liu *et al.*[23]	$Mg_3(Sb, Bi)_2$	8.97	Yin *et al.*[24]
PbTe	1.88	Xu *et al.*[25]	$Mg_2Si_{0.3}Sn_{0.7}$	7.45	Cheng *et al.*[26]
PbSe	1.76	Jiang *et al.*[27]	$Mg_2Sn_{0.75}Ge_{0.25}$	8.02	Wu *et al.*[28]
Skutterudites	1.31	Tang *et al.*[29]	PbTe	4.01	Deng *et al.*[30]
half-Heusler	1.03	Ren *et al.*[31]	$PbSnS_2$	2.65	Zhan *et al.*[32]
SiGe	1.14	Zebarjadi *et al.*[33]	GeSe	6.13	Lou *et al.*[34]
Bi_2Te_3	0.92	Liu *et al.*[35]	n-type Bi_2Te_3	4.21	Lou *et al.*[36]
$Mg_3(Sb,Bi)_2$	1.85	Liu *et al.*[7]	GeTe	8.91	Sarkar *et al.*[37]
x = 0.02-T20	1.95	This work	MgAgSb	8.45	Li *et al.*[38]
-	-	-	$AgSbTe_2$	9.80	Roychowdhury *et al.*[39]

Supplementary Table 3. Polar coupling constant for Mg_3Sb_2 and related parameters determined from phonon dispersion and literature[14].

Materials	Mg_3Sb_2
ε_∞	14.7
ε_s	31.3
ω_{LO}	7.2 THz
m^*	1.44 m_e
α_{PO}	0.92

Supplementary Table 4. Fitted parameters obtained from the Debye-Einstein model for low-temperature heat capacity.

Materials	$x = 0$	$x = 0.02$-T20
γ (mJ mol^{-1} K^{-2})	0.23	0.084
B (mJ mol^{-1} K^{-4})	0.92	0.96
Θ_D (K)	219	216
Θ_{E1} (K)	45.10	42.69
Θ_{E2} (K)	103.51	99.94
Θ_{E3} (K)	300	300

Supplementary Table 5. Fitted parameters of the Debye-Callaway model for the studied samples.

Materials	$x = 0$	$x = 0.02$	$x = 0.02$-T20
Composition	$Mg_3Sb_{1.5}Bi_{0.49}Te_{0.01}$	$Mg_3Sb_{1.48}Sn_{0.02}Bi_{0.49}Te_{0.01}$	$Mg_3Sb_{1.48}Sn_{0.02}Bi_{0.49}Te_{0.01}$
n	1.129	1.112	1.104
m	0.753	1.000	1.357
U	0.5	0.5	0.5
Γ_{MF}	0.218	0.236	0.236
Γ_{SF}	0.234	0.32	0.677

Supplementary Table 6. The data of $1/\kappa_{lat}$ and μ of $Mg_3(Sb,Bi)_2$-based materials compiled from literature.

Materials	$1/\kappa_{lat}$ ($W^{-1}mK$)	μ ($cm^2V^{-1}s^{-1}$)	Ref.	Materials	$1/\kappa_{lat}$ ($W^{-1}mK$)	μ ($cm^2V^{-1}s^{-1}$)	Ref
Cu&Te codoping	0.98<br>1.18<br>1.31<br>1.31<br>1.15	60<br>117.59<br>134.39<br>149.12<br>173	Liu *et al.*[7]	Mn&Te codoping	1.00<br>1.20<br>1.21<br>1.18<br>1.15	19.48<br>52.65<br>58.19<br>43.61<br>39.62	Chen *et al.*[12]
Gd doping	1.25<br>1.34<br>1.36<br>1.57<br>1.47<br>1.50	107.13<br>129.46<br>146.89<br>122.60<br>64.47<br>97.73	Lei *et al.*[18]	Y doping	1.23<br>1.09<br>1.10<br>1.11<br>1.19<br>1.41<br>1.15<br>1.08<br>1.39<br>1.08	122<br>126<br>136<br>125<br>115<br>120<br>120<br>126<br>115<br>95	Shi *et al.*[20]
Se doping	1.34<br>1.35<br>1.42<br>1.19<br>1.26	75.75<br>64.70<br>63.29<br>60.43<br>62.23	Zhang *et al.*[22]	TM&Te codoping	1.29<br>1.36<br>1.36<br>1.33	124<br>148.88<br>159.76<br>141.36	Jiang *et al.*[13]
Y&Se codoping	0.96<br>0.80<br>1.44<br>1.03	35.92<br>32.17<br>31.51<br>31.37	Liang *et al.*[40]	Ho doping	0.83<br>0.89<br>1.01<br>1.17	16.14<br>17.48<br>28.67<br>51.01	Chen *et al.*[15]
Ti&Te codoping	1.28<br>1.37<br>1.37<br>1.38<br>1.33	124.64<br>141.44<br>147.26<br>155.66<br>144.64	Jiang *et al.*[17]	TiO_{2-n}&Te codoping	1.061<br>1.18<br>1.28<br>1.21<br>1.21	140.33<br>155.69<br>166.52<br>149.90<br>176.31	Li *et al.*[41]
Y doping	1.56<br>1.92<br>2.04<br>2.27	1.56<br>1.92<br>2.04<br>2.27	Li *et al.*[42]	Al&Te codoping	1.00<br>1.04<br>1.09<br>1.12<br>1.12<br>1.06	29.67<br>28.17<br>28.36<br>28.22<br>27.58<br>26.69	Zhang *et al.*[43]
Co/Mn&Se codoping	1.12<br>1.27<br>1.23<br>1.16	26.20<br>122.26<br>126.45<br>138.61	Zhang *et al.*[11]	Co/Mn&Se codoping	1.77<br>1.75<br>1.66	88.52<br>86.09<br>89.66	Zhang *et al.*[11]

Supplementary Table 7. The average *zT* data of $Mg_3(Sb,Bi)_2$ and Bi_2Te_3 materials compiled from literature.

Samples	zT_{av} (300–723K)	Ref	Samples	zT_{av} (300–573K)	Ref
Te doping	0.95	Tamaki *et al.*[44]	Mn&Te codoping	1.02	Chen *et al.*[12]
Tm&Te codoping	1.08	Zhang *et al.*[45]	Cu&Te codoping	1.06	Liu *et al.*[7]
Nb&Te codoping	1.14	Shuai *et al.*[10]	Y doping	1.068	Pei *et al.*[20]
Cu&Te codoping	1.24	Liu *et al.*[7]	Te doping	0.91	Yang *et al.*[46]
Mn&Te codoping	1.25	Chen *et al.*[12]	Y doping	1.1	Ying *et al.*[47]
Y doping	1.30	Pei *et al.*[20]	Mg_2Cu&Te codoping	1.22	Lei *et al.*[48]
TiO_{2-n}&Te added	1.38	Li *et al.*[41]	n-type Bi_2Te_3	1.16	Liu *et al.*[35]

Supplementary Table 8. Measured longitudinal sound velocity (v_l), transverse sound velocity (v_t), average sound velocity (v), density (d), and derived elastic parameters of the samples.

Samples	v_l (m/s)	v_t (m/s)	v (m/s)	d (g/cm^3)	E (GPa)	G (GPa)	B (GPa)	Poisson ratio	γ
x = 0	3781	1842	2069	4.473	40.80	15.17	43.72	0.34	2.09
x = 0.01	3793	1844	2072	4.458	40.79	15.16	43.92	0.35	2.09
x = 0.02	3790	1837	2064	4.46	40.53	15.05	44.01	0.35	2.10
x = 0.02-T20	3758	1841	2067	4.456	40.53	15.10	42.78	0.34	2.07
x = 0.04	3778	1831	2058	4.451	40.19	14.93	43.64	0.35	2.10

x = 0.04-T20	3766	1825	2051	4.457	39.98	14.85	43.40	0.35	2.10

1. Agne MT, Imasato K, Anand S *et al.* Heat capacity of Mg_3Sb_2, Mg_3Bi_2, and their alloys at high temperature. *Mater Today Phys* 2018; **6**: 83–88.
2. Togo A, Tanaka I. First principles phonon calculations in materials science. *Scripta Mater* 2015; **108**: 1–5.
3. Kresse G, Furthmüller J. Efficient iterative schemes for *ab initio* total-energy calculations using a plane-wave basis set. *Phys Rev B* 1996; **54**: 11169–11186.
4. Petersen A, Bhattacharya S, Tritt TM *et al.* Critical analysis of lattice thermal conductivity of half-Heusler alloys using variations of Callaway model. *J Appl Phys* 2015; **117**: 035706.
5. Gurunathan R, Hanus R, Snyder GJ. Alloy scattering of phonons. *Mater Horizons* 2020; **7**: 1452–1456.
6. Wang L, Zhang W, Back SY *et al.* High-performance Mg_3Sb_2-based thermoelectrics with reduced structural disorder and microstructure evolution. *Nat Commun* 2024; **15**: 6800.
7. Liu Z, Sato N, Gao W *et al.* Demonstration of ultrahigh thermoelectric efficiency of ∼7.3% in Mg_3Sb_2/MgAgSb module for low-temperature energy harvesting. *Joule* 2021; **5**: 1196–1208.
8. Wang L, Sato N, Peng Y et al. Realizing high thermoelectric performance in n-type $Mg_3(Sb, Bi)_2$-based materials via synergetic Mo addition and Sb-Bi ratio refining. *Adv Energy Mater* 2023; **13**: 2301667.
9. Zhang J, Song L, Borup KA *et al.* New insight on tuning electrical transport properties via chalcogen doping in n-type Mg_3Sb_2-based thermoelectric materials. *Adv Energy Mater* 2018; **8**: 1702776.
10. Shuai J, Mao J, Song S *et al.* Tuning the carrier scattering mechanism to effectively improve the thermoelectric properties. *Energy Environ Sci* 2017; **10**: 799–807.
11. Zhang F, Chen C, Yao H *et al.* High□performance n□type Mg_3Sb_2 towards thermoelectric application near room temperature. *Adv Funct Mater* 2019; **30**: 1906143.
12. Chen X, Wu H, Cui J *et al.* Extraordinary thermoelectric performance in n-type manganese doped Mg_3Sb_2 Zintl: High band degeneracy, tuned carrier scattering mechanism and hierarchical microstructure. *Nano Energy* 2018; **52**: 246–255.
13. Jiang F, Feng T, Zhu Y *et al.* Extraordinary thermoelectric performance, thermal stability and mechanical properties of n-type $Mg_3Sb_{1.5}Bi_{0.5}$ through multi-dopants at interstitial site. *Mater Today Phys* 2022; **27**: 100835.
14. Mao J, Shuai J, Song S *et al.* Manipulation of ionized impurity scattering for achieving high thermoelectric performance in n-type Mg_3Sb_2-based materials. *Proc Natl Acad Sci USA* 2017; **114**: 10548–10553.
15. Chen S, Wei J, Kang Z *et al.* Enhancement of thermoelectric performance in $Mg_3(Sb,Bi)_2$ through engineered lattice strain and controlled carrier scattering. *Chem Eng J* 2024; **490**: 151404.
16. Shuai J, Ge B, Mao J *et al.* Significant role of Mg stoichiometry in designing high thermoelectric

performance for $Mg_3(Sb,Bi)_2$-based n-type Zintls. *J Am Chem Soc* 2018; **140**: 1910–1915.

17. Jiang F, Wu X, Zhu Y *et al.* Boosting room-temperature thermoelectric performance of $Mg_3Sb_{1.5}Bi_{0.5}$ material through breaking the contradiction between carrier concentration and carrier mobility. *Acta Mater* 2024; **265**: 119636.

18. Lei J, Wuliji H, Zhao K *et al.* Efficient lanthanide Gd doping promoting the thermoelectric performance of Mg_3Sb_2-based materials. *J Mater Chem A* 2021; **9**: 25944–25953.

19. Li J, Jia F, Zhang S *et al.* The manipulation of substitutional defects for realizing high thermoelectric performance in Mg_3Sb_2-based Zintl compounds. *J Mater Chem A* 2019; **7**: 19316–19323.

20. Shi X, Zhao T, Zhang X *et al.* Extraordinary n-type Mg_3SbBi thermoelectrics enabled by yttrium doping. *Adv Mater* 2019; **31**: 1903387.

21. Zhang J, Song L, Pedersen SH *et al.* Discovery of high-performance low-cost n-type Mg_3Sb_2-based thermoelectric materials with multi-valley conduction bands. *Nat Commun* 2017; **8**: 13901.

22. Zhang J, Song L, Mamakhel A *et al.* High-performance low-cost n-type Se-doped Mg_3Sb_2-based Zintl compounds for thermoelectric application. *Chem Mater* 2017; **29**: 5371–5383.

23. Liu W, Tan X, Yin K *et al.* Convergence of conduction bands as a means of enhancing thermoelectric performance of n-type $Mg_2Si_{1-x}Sn_x$ solid solutions. *Phys Rev Lett* 2012; **108**: 166601.

24. Yin L, Chen C, Zhang F *et al.* Reliable N-type $Mg_{3.2}Sb_{1.5}Bi_{0.49}Te_{0.01}$/304 stainless steel junction for thermoelectric applications. *Acta Mater* 2020; **198**: 25–34.

25. Xu P, Zhao W, Liu X *et al.* Dramatic enhancement of thermoelectric performance in PbTe by unconventional grain shrinking in the sintering process. *Adv Mater* 2022; **34**: e2202949.

26. Cheng K, Bu Z, Tang J *et al.* Efficient $Mg_2Si_{0.3}Sn_{0.7}$ thermoelectrics demonstrated for recovering heat of about 600 K. *Mater Today Phys* 2022; **28**: 100887.

27. Jiang B, Yu Y, Cui J *et al.* High-entropy-stabilized chalcogenides with high thermoelectric performance. *Science* 2021; **371**: 830–834.

28. Wu X, Lin Y, Liu C *et al.* Interface engineering boosting high power density and conversion efficiency in $Mg_2Sn_{0.75}Ge_{0.25}$□based thermoelectric devices. *Adv Energy Mater* 2023; **13**: 2301350.

29. Tang Y, Gibbs ZM, Agapito LA *et al.* Convergence of multi-valley bands as the electronic origin of high thermoelectric performance in $CoSb_3$ skutterudites. *Nat Mater* 2015; **14**: 1223–1228.

30. Deng P-Y, Wang K-K, Sung H-Y *et al.* Liquid-like copper chalcogenide modulates electron donors in high-performance n-type PbTe thermoelectrics. *Cell Rep Phys Sci* 2023; **4**: 101413.

31. Ren W, Xue W, Guo S *et al.* Vacancy-mediated anomalous phononic and electronic transport in defective half-Heusler ZrNiBi. *Nat Commun* 2023; **14**: 4722.

32. Zhan S, Hong T, Qin B *et al.* Realizing high-ranged thermoelectric performance in $PbSnS_2$ crystals. *Nat Commun* 2022; **13**: 5937.

33. Zebarjadi M, Joshi G, Zhu G *et al.* Power factor enhancement by modulation doping in bulk nanocomposites. *Nano Lett* 2011; **11**: 2225–2230.

34. Luo H, Shi X-L, Liu Y *et al.* Metavalent alloying and vacancy engineering enable state-of-the-art cubic GeSe thermoelectrics. *Nat Commun* 2025; **16**: 3136.

35. Liu D, Bai S, Wen Y *et al.* Lattice plainification and band engineering lead to high thermoelectric cooling and power generation in n-type Bi_2Te_3 with mass production. *Natl Sci Rev* 2025; **12**: nwae448.

36. Lou L-Y, Yang J, Zhu Y-K *et al.* Tunable electrical conductivity and simultaneously enhanced thermoelectric and mechanical properties in n-type Bi_2Te_3. *Adv Sci* 2022; **9**: 2203250.

37. Sarkar D, Das S, Taneja V *et al.* Glassy thermal transport triggers ultra-high thermoelectric performance in GeTe. *Adv Mater* 2025; **37**: e2417561.

38. Li A, Wang L, Li J *et al.* Global softening to manipulate sound velocity for reliable high-performance MgAgSb thermoelectrics. *Energy Environ Sci* 2024; **17**: 8810–8819.

39. Roychowdhury S, Ghosh T, Arora R *et al.* Enhanced atomic ordering leads to high thermoelectric performance in $AgSbTe_2$. *Science* 2021; **371**: 722–727.

40. Liang J-S, Shi X-L, Peng Y *et al.* Synergistic effect of band and nanostructure engineering on the boosted thermoelectric performance of n□type $Mg_{3+\delta}(Sb, Bi)_2$ Zintls. *Adv Energy Mater* 2022; **12**: 2201086.

41. Li J-W, Gao H, Han Z *et al.* In situ engineering of grain boundary phase toward superior thermoelectric performance in $Mg_3(Sb,Bi)_2$. *Adv Mater* 2025; **37**: e2503665.

42. Li J-W, Liu W, Xu W *et al.* Bi-deficiency leading to high-performance in $Mg_3(Sb,Bi)_2$-based thermoelectric materials. *Adv Mater* 2023; **35**: e2209119.

43. Zhang Q, Li H, Chauhan NS *et al.* Insights into enhanced thermoelectric performance of the n-type Mg_3Sb_2-based materials by amphoteric Al doping. *Mater. Today Energy* 2024; **44**: 101656.

44. Tamaki H, Sato HK, Kanno T. Isotropic conduction network and defect chemistry in $Mg_{3+\delta}Sb_2$-based layered Zintl compounds with high thermoelectric performance. *Adv Mater* 2016; **28**: 10182–10187.

45. Zhang J, Song L, Iversen BB. Probing efficient n-type lanthanide dopants for Mg_3Sb_2 thermoelectrics. *Adv Sci* 2020; **7**: 2002867.

46. Yang J, Li G, Zhu H *et al.* Next-generation thermoelectric cooling modules based on high-performance $Mg_3(Bi,Sb)_2$ material. *Joule* 2022; **6**: 193–204.

47. Ying P, Wilkens L, Reith H *et al.* A robust thermoelectric module based on $MgAgSb/Mg_3(Sb,Bi)_2$ with a conversion efficiency of 8.5% and a maximum cooling of 72 K. *Energy Environ Sci* 2022; **15**: 2557–2566.

48. Lei J, Zhao K, Liao J *et al.* Approaching crystal's limit of thermoelectrics by nano-sintering-aid at grain boundaries. *Nat Commun* 2024; **15**: 6588.
